# Mutation of CFAP57, a protein required for the asymmetric targeting of a subset of inner dynein arms in *Chlamydomonas*, causes primary ciliary dyskinesia

**DOI:** 10.1371/journal.pgen.1008691

**Published:** 2020-08-07

**Authors:** Ximena M. Bustamante-Marin, Amjad Horani, Mihaela Stoyanova, Wu-Lin Charng, Mathieu Bottier, Patrick R. Sears, Wei-Ning Yin, Leigh Anne Daniels, Hailey Bowen, Donald F. Conrad, Michael R. Knowles, Lawrence E. Ostrowski, Maimoona A. Zariwala, Susan K. Dutcher

**Affiliations:** 1 Department of Medicine, Marsico Lung Institute, University of North Carolina, Chapel Hill, North Carolina, United States of America; 2 Department of Pediatrics, Washington University School of Medicine, St. Louis, Missouri, United States of America; 3 Department of Genetics, Washington University School of Medicine, St. Louis, Missouri, United States of America; 4 Department of Neurology, Washington University School of Medicine, St. Louis, Missouri, United States of America; 5 Department of Mechanical Engineering, Washington University, St. Louis, Missouri, United States of America; 6 Division of Genetics, Oregon National Primate Research Center, Oregon Health & Science University, Beaverton, Oregon, United States of America; 7 Department of Molecular and Medical Genetics, Oregon Health & Science University, Portland, Oregon, United States of America; 8 Department of Pathology and Laboratory Medicine and the Marsico Lung Institute, University of North Carolina, Chapel Hill, North Carolina, United States of America; Rutgers University, UNITED STATES

## Abstract

Primary ciliary dyskinesia (PCD) is characterized by chronic airway disease, reduced fertility, and randomization of the left/right body axis. It is caused by defects of motile cilia and sperm flagella. We screened a cohort of affected individuals that lack an obvious axonemal defect for pathogenic variants using whole exome capture, next generation sequencing, and bioinformatic analysis assuming an autosomal recessive trait. We identified one subject with an apparently homozygous nonsense variant [(c.1762C>T), p.(Arg588*)] in the uncharacterized *CFAP57* gene. Interestingly, the variant results in the skipping of exon 11 (58 amino acids), which may be due to disruption of an exonic splicing enhancer. In normal human nasal epithelial cells, CFAP57 localizes throughout the ciliary axoneme. Nasal cells from the PCD patient express a shorter, mutant version of *CFAP57* and the protein is not incorporated into the axoneme. The missing 58 amino acids include portions of WD repeats that may be important for loading onto the intraflagellar transport (IFT) complexes for transport or docking onto the axoneme. A reduced beat frequency and an alteration in ciliary waveform was observed. Knockdown of *CFAP57* in human tracheobronchial epithelial cells (hTECs) recapitulates these findings. Phylogenetic analysis showed that CFAP57 is highly conserved in organisms that assemble motile cilia. *CFAP57* is allelic with the *BOP2/IDA8/FAP57* gene identified previously in *Chlamydomonas reinhardtii*. Two independent, insertional *fap57 Chlamydomonas* mutant strains show reduced swimming velocity and altered waveforms. Tandem mass tag (TMT) mass spectroscopy shows that FAP57 is missing, and the “g” inner dyneins (DHC7 and DHC3) and the “d” inner dynein (DHC2) are reduced, but the FAP57 paralog FBB7 is increased. Together, our data identify a homozygous variant in *CFAP57* that causes PCD that is likely due to a defect in the inner dynein arm assembly process.

## Author summary

Primary ciliary dyskinesia (PCD) is a rare genetic disease that affects the function of motile cilia. The estimated incidence is about 1 in 15,000 births, but diagnosis can be difficult since it is genetically heterogenous. At present, about 30% of PCD patients lack a genetic diagnosis. By applying whole exome sequencing and bioinformatic analysis, we identified a variant in an uncharacterized gene, *CFAP57*, in a subject previously diagnosed with PCD. This is the first reported example of PCD caused by a mutation that likely only affects a subset of the inner dynein arms. These findings demonstrate the effectiveness of whole exome sequencing and bioinformatic analysis for identifying pathogenic variants in rare genetic diseases like PCD, expand our understanding of how dynein arms are positioned during cilia assembly, and identify a variant in *CFAP57* that causes PCD.

## Introduction

Motile cilia are complex organelles that project from the surface of cells and are essential for propelling fluids in the airways, ventricles and fallopian tubes or for providing cell locomotion as in sperm or *Chlamydomonas*. The microtubule-based axoneme shows the classic structure of 9 outer doublet and 2 singlet central pair microtubules, which is conserved throughout the eukaryotic lineage. Motility comes from the coordinated activity of inner and outer dynein arms (IDA and ODA, respectively) that are attached to the A tubule of the microtubule doublets. Defects in motile cilia cause primary ciliary dyskinesia (PCD; MIM:244400), which is a genetically and phenotypically heterogeneous disorder that is characterized by chronic and debilitating respiratory disease, and is frequently accompanied by laterality defects (~50% of patients) due to abnormal left-right body asymmetry [1, 2]. Usually PCD is an autosomal recessive trait, but autosomal dominant and X-linked traits have been reported [3, 4]. The most common genetic variants that cause PCD affect components of the ODA, which include, for example, *DNAH5*, *DNAI1*, and *DNAH11* [5–8]. Other groups of PCD associated genes encode ODA-docking complex components (*CCDC114*, *CCDC151*, *TTC25*, and *ARMC4*) [9–14], or genes that code for proteins that play a role in the cytoplasmic assembly of dynein arms, (*DNAAF1-3*, *DYX1C1*, *HEATR2*, *LRRC6*, *PIH1D3*, *SPAG1*, *ZMYND10*) [3, 15–24]. In addition, mutations in two genes, *CCNO* [25] and *MCIDAS* [26], cause a PCD-like phenotype by greatly reducing the number of motile cilia. Although genetic variants that cause PCD have been identified in over 40 genes [27–37], there are individuals with confirmed clinical features of PCD but normal axonemal structure as determined by transmission electron microscopy, and the genetic basis of disease remains unknown in about 30% of patients. This diagnostic dilemma is likely to require multiple techniques and approaches. With the continual improvement in sequencing techniques and bioinformatic analysis, whole exome sequencing provides a powerful tool to confirm the diagnosis of PCD in these difficult cases.

Many of the genes identified in PCD patients have orthologs in the unicellular green alga, *Chlamydomonas reinhardtii* [17, 38–42]. *Chlamydomonas* has provided a wide range of candidate genes for PCD [40, 43] as well as providing a model system for probing molecular mechanisms of ciliary assembly and function. In *Chlamydomonas*, the IDAs play a crucial role in determining axonemal waveform and are more biochemically complex than the ODAs. *Chlamydomonas* has six single headed IDA (a, b, c, d, e, and g) and one double headed IDA (I1/f) that span the 96 nm repeat, based on biochemical analysis [44, 45], genetic analysis [46] and most recently by cryo-EM tomography using mutant strains affecting these proteins [47, 48]. In addition, there are three unique minor IDAs that are only found in the proximal regions and replace the major IDAs; these include *DHC3*, *DHC4*, and *DHC11* [49]. Mutations in IDAs are difficult to detect by transmission electron microscopy (TEM) cross sections since there are 7 dynein arms in 96 nm, and TEM sections are often 60 nm. Thus, TEM lacks the resolution necessary to detect changes in individual IDAs, as a single IDA can be obscured by the other IDAs in the section.

The *Chlamydomonas BOP2* locus was first identified as a suppressor of the swimming defect of the *pf10* mutant [50]. Double mutant analysis, using IDA and ODA mutants, suggested that the mutation in the *BOP2* locus affects only the IDAs. Averaging of longitudinal sections from electron microscopy of the *bop2-1* mutant showed that a subset of dynein arms were missing on only some of the microtubule doublets [51]. Specifically, reduced intensity on doublets 5, 6, and 8 was observed, while doublet 9 showed an intermediate loss of intensity [51]. Recent cryo-EM tomography confirmed this radial asymmetry and showed that the *BOP2* locus encodes FAP57, which forms an extended structure that interconnects multiple IDAs with the largest effect on dynein d and g [52]. This locus has multiple names (*BOP2/IDA8/FAP57/WDR65*) [50–52], but we will call it *FAP57* except when referring to the original *bop2-1* allele. To date, no pathogenic variants that cause PCD have been identified in genes that affect only the IDA complexes, while several mutations have been identified that cause both axonemal disorganization and absence of IDA [53].

In an ongoing effort, we have performed whole exome capture sequencing on more than 400 unrelated cases in order to identify genetic causes of PCD. Within this cohort, there were 99 unrelated cases with clinical features of PCD that include chronic oto-sino-pulmonary symptoms, and low levels of nasal nitric oxide who presented with no obvious axonemal defects by TEM. Using a population sampling probability algorithm (PSAP) to analyze exome sequencing data [54], we identified an apparently homozygous stop-gain variant in ciliary and flagella associated protein 57 (*CFAP57/WDR65*; MIM: 614259; NM_001195831.2) the human ortholog of *FAP57*. The individual has classical symptoms of PCD that included bronchiectasis, neonatal respiratory distress, otitis media, and sinusitis. Our data suggests that homozygosity of a pathogenic variant of *CFAP57* causes PCD, and our results using *Chlamydomonas* suggest that this is likely due to a failure to assemble a subset of inner dynein arms. *CFAP57* should therefore be considered a candidate gene in cases of PCD with apparently normal axonemal structure by TEM and significantly reduced CBF.

## Results

### Whole exome capture and sequencing identifies a *CFAP57* pathogenic variant in a PCD subject

**Fig 1:**
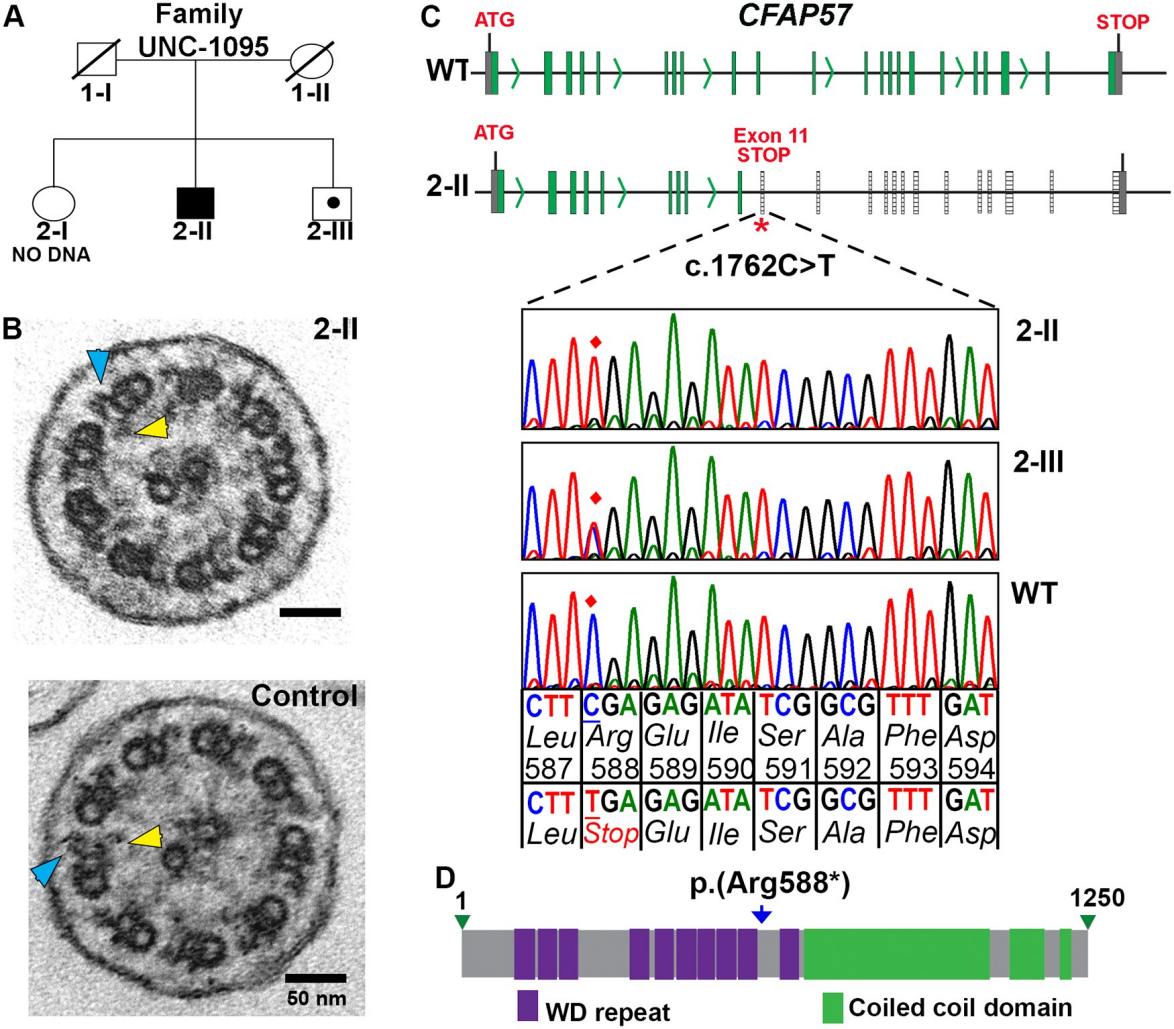
Segregation analysis of pathogenic variant in *CFAP57* found in family UNC-1095. (A) Pedigree analysis of family UNC-1095. Males and females are designated by squares and circles, respectively. Filled symbols indicate the PCD affected individual (Proband 2-II). Carrier status is indicated with a dot within the symbols. Symbols with strike-through indicate deceased individual and DNA was not available. (B) Transmission electron micrograph of axonemal cross section of nasal epithelium from proband 2-II and unrelated control showing the central pair surrounded by nine microtubule doublets. Outer dynein arms (blue arrowhead) and inner dynein arms (yellow arrowhead) project from each doublet normally. (C) Whole exome sequencing of proband 2-II identified an apparently homozygous pathogenic variant c.1762C>T p.(Arg588*) in exon 11 of *CFAP57* (NM_001195831.2) on Chr.1p34.2. The top panel shows normal exon-intron structure and the bottom panel shows the location of the apparently homozygous pathogenic variant c.1762C>T in exon 11 that introduces a premature stop codon. The position of the pathogenic allele is highlighted by the red diamond in the electropherograms. (D) Predicted diagram of CFAP57 of the shorter but curated 1250 amino acid isoform of CFAP57 (UNIPROT Q96MR6) showing 10 WD repeats and 3 coiled coil domains. The predicted location of the mutations p.(Arg588*) is indicated by the blue arrow.

We identified a 38-year-old male from family UNC-1095 (Fig 1A) with a typical PCD phenotype that includes neonatal respiratory distress, otitis media, sinusitis, bronchiectasis, and a low rate of nasal nitric oxide production (40 nl/min; cutoff 77 nl/min) [55]; however no axonemal defect was detected by TEM (Fig 1B). DNA from the research subject, who had been previously screened and found negative for mutations in genes known to be associated with PCD, was whole exome sequenced using the IDT capture reagent [56]. We analyzed the resulting genotype data using the population sampling probability algorithm (PSAP), a statistical framework for assessing the significance of variants from n = 1 cases of rare genetic disease [54] (S1 Table), assuming an autosomal recessive trait. A new candidate gene, *CFAP57*, was identified and confirmed by Sanger sequencing that revealed an apparently homozygous nonsense variant [(c.1762C>T) p.(Arg588*)] in exon 11 of *CFAP57* (Fig 1C), which is predicted to introduce a premature stop codon and result in complete loss of function of CFAP57 (Fig 1D). One unaffected sibling is a carrier of the pathogenic variant, consistent with an autosomal recessive pattern of inheritance. This variant is listed as rs369556067 in dbSNP (http://www.ncbi.nlm.NIH.gov/snp/) and is represented at a very low allele frequency (3 alleles out of 250650 alleles) in the genome aggregation database (https://gnomad.broadinstitute.org). There are multiple transcripts reported for *CFAP57*, however full length *CFAP57* encode 1283 amino acids resulting in an ~145 kDa protein that is predicted to contain 10 WD repeats along with 3 predicted coiled-coil domains (Fig 1D). PSAP analysis identified the homozygous nonsense variant in *CFAP57* as the second most deleterious change in the subject’s genome, following a homozygous missense change in *CD2*, a T-cell surface antigen. Variants in the latter do not explain the subject’s symptoms (S1 Table).

### Expression of *CFAP57* in ciliated human airway epithelial cells

**Fig 2:**
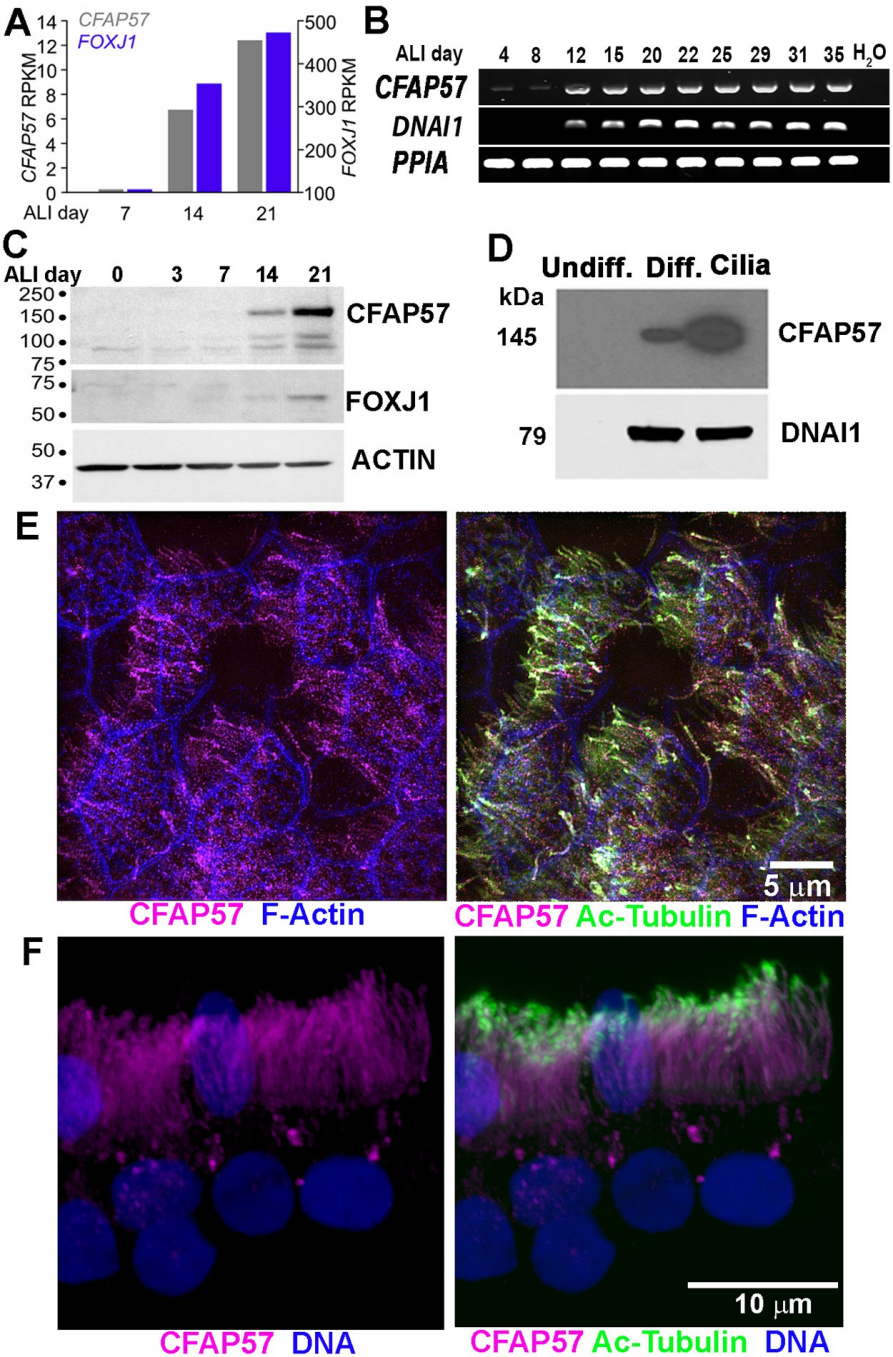
Expression and localization of *CFAP57* in human airway epithelial cells. (A and B) *CFAP57* mRNA in normal hTEC cells. Total RNA was extracted at the indicated day of ALI culture. (A) Levels of mRNA for *CFAP57* and *FOXJ1* rise during ALI culture from RNAseq experiments. (B) Transcript levels of *CFAP57* increase, during airway cell differentiation, in parallel to the expression of *DNAI1*, a ciliary specific gene. *PPIA* was used as housekeeping gene control. (C) Immunoblots showing increased levels of CFAP57 as ciliated airway cells underwent differentiation along with increased levels of FOXJ1. (D) Immunoblot showing enrichment of CFAP57 in isolated ciliary axonemes (cilia) compared to differentiated cells (Diff) extract. CFAP57 was absent in protein extract from undifferentiated cells (Undiff). DNAI1 was used as a loading control for presence of cilia. Predicted sizes are shown. (E and F) The localization of CFAP57 (magenta) to the cilia was confirmed by immunofluorescence in (E) whole ALI culture and in (F) ciliated cells.

We sought to determine the expression and localization of *CFAP57* in airway cells. Primary human tracheal epithelial (hTEC) cells were cultured with air-liquid interface conditions as previously described [57]. Under these conditions, the cells first proliferate as an undifferentiated monolayer and then undergo differentiation to form ciliated cells. In hTEC cells, the *CFAP57* mRNA increases in parallel to the amount of *FOXJ1* mRNA, a key gene that drives ciliogenesis (Fig 2A) and to the *DNAI1* (dynein axonemal intermediate chain 1) mRNA, a known ciliary specific gene downstream of the expression of *FOXJ1* (Fig 2B). Similarly, the level of CFAP57 protein increases as ciliated airway cells underwent differentiation, as determined by increased levels of FOXJ1 detected by immunoblot (Fig 2C). The CFAP57 protein is strongly enriched in samples of detergent isolated ciliary axonemes (Fig 2D). Immunofluorescent staining of CFAP57 in both intact hTEC cultures (Fig 2E) and isolated ciliated cells from cultures (Fig 2F) and human tracheas (S1 Fig) demonstrate strong positive reactivity throughout the length of the ciliary axoneme. These results demonstrate that CFAP57 is an axonemal protein as suggested previously by proteomic analysis of both human and *Chlamydomonas* axonemes [43, 58].

### A pathogenic variant of *CFAP57* causes defective ciliary beating

**Fig 3:**
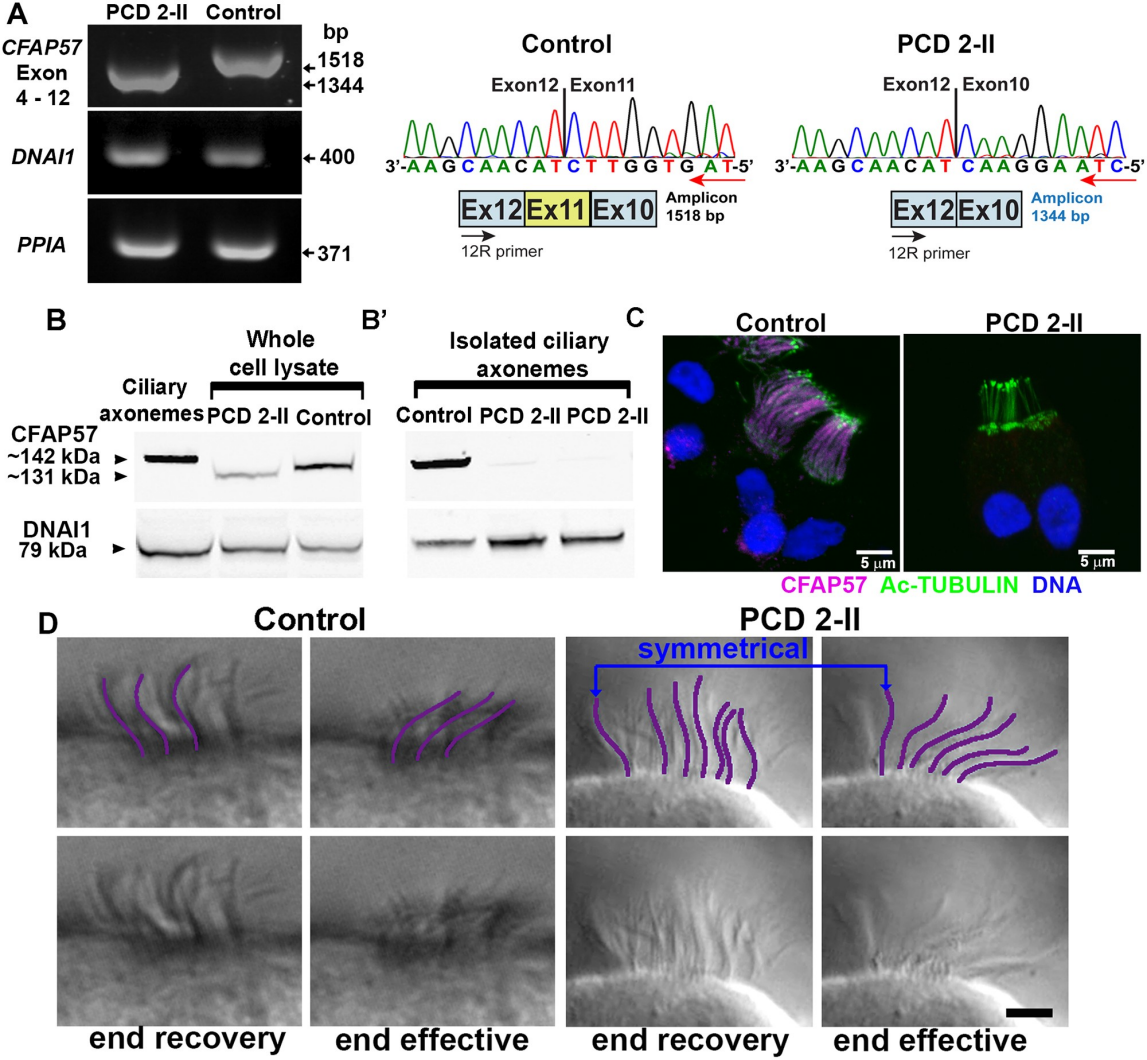
The pathogenic variant in *CFAP57* results in a shorter protein that does not assemble into the axoneme and results in an altered ciliary beat. (A) (*left*) RT-PCR analysis of the subject mRNA. (*right*) Sequence analysis of PCR product demonstrates the skipping of exon 11 (r.1758_1931del; p.Ile586_Lys643del). Sequences are shown in the reverse complement direction. (B) Immunoblotting demonstrates the presence of a mutant form of CFAP57 protein in nasal cell lysates from the PCD subject and (B’) the lack of CFAP57 in the isolated ciliary axonemal fraction. Approximate sizes based on migration of size standards are shown. (C) Immunofluorescence of cultured nasal epithelial cells shows the absence of CFAP57 (magenta) in ciliated cells from the PCD 2-II subject compared to control cells. (D) High-resolution videos of control and PCD 2-II ciliated cells were examined. Compared to control cells, PCD 2-II cells show heterogeneous waveforms. In some cilia the waveform was symmetrical (blue arrows, abnormal) while in other cilia the wave form showed a normal pattern. Scale bar = 2 μm.

To characterize the effects of the genetic variant in *CFAP57* on the function of motile cilia, we obtained human nasal epithelial (HNE) cells from the PCD subject and unrelated healthy controls. Cells were expanded in culture as conditionally reprogrammed cells (CRCs); a cell culture methodology that enhances cell growth and lifespan while preserving cell-of origin-functionality [59], then allowed to differentiate using air-liquid interface cultures as previously described [33]. RT-PCR analysis with primers that flanked exon 11 result in the amplification of a shorter PCR product from the subject compared to controls (Fig 3A and S2 Fig). Surprisingly, sequencing of the amplified product revealed that exon 11 is missing in the sample from the subject (Fig 3A), which suggests that the sequence change may disrupt an exonic splice enhancer (ESE) [60]. Immunoblotting demonstrates the presence of a smaller protein in cell lysates from the PCD subject (Fig 3B), consistent with an in-frame loss of 58 amino acids that arises from the skipping of exon 11. Importantly, the mutant protein fails to assemble in the ciliary axoneme, as evidenced by both immunoblotting of detergent isolated ciliary axonemes (Fig 3B’) and immunostaining of isolated ciliated cells (Fig 3C). In contrast, immunofluorescent intensities for the ODA protein DNAH5 and the radial spoke component RSPH1 are unchanged between PCD and control cells (S3 Fig), in agreement with the TEM analysis that showed no obvious axonemal structural defect (Fig 1B). In addition, the fluorescent intensity of the IDA component, DNALI1, was not obviously different in cells obtained from the PCD subject compared to control cells (S3 Fig).

The absence of CFAP57 in the axoneme resulted in a significant reduction of ciliary beat frequency (CBF) in the PCD cells. Compared to control cells (CBF = 16.98 +/- 4.35 Hz, n = 11), CBF in the PCD cells is reduced ~ 30% (10.75 +/- 1.54 Hz) (n = 16; p<0.0007). Using high resolution video microscopy, we performed a detailed analysis of the ciliary waveform. In the PCD cells the waveform is altered. Although in some ciliated cells the waveform appeared normal, in most, the waveform appeared symmetric, with no clear effective and recovery stroke (Fig 3D and S1 and S2 Videos). An analysis of maximum displacement from linearity [61] between control (0.53 +/- 0.44 μm) and PCD cells (0.30 +/- 0.10 μm) is not significant (p = 0.3; n = 4), which indicates that the PCD cells maintain a planar beat. Thus, the PCD cells exhibit a reduced CBF and an altered waveform.

### Silencing of *CFAP57* in hTEC reduces ciliary motility

**Fig 4:**
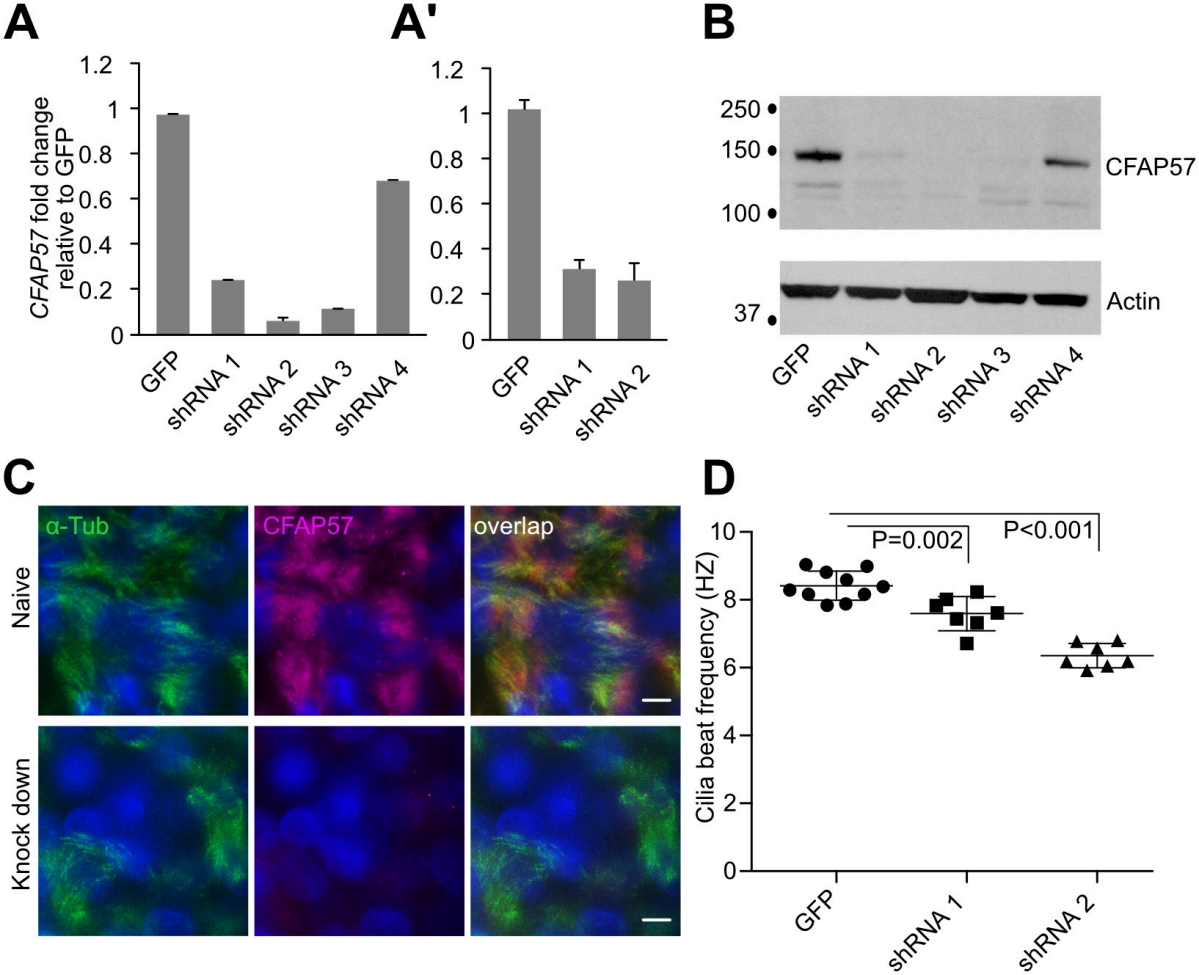
*CFAP57* silencing in cultured airway cells causes decreased ciliary motility. (A) The fold changes in *CFAP57* after transduction with four specific *CFAP57* shRNA sequences normalized to the level in the GFP hTEC cells. shRNA4 has only a weak effect on mRNA levels (n = 3 technical replicates). (A’) Cumulative data of 3 separate experiments using 3 different donors of primary hTEC (n = 3 biological replicates). (B) Immunoblot on the shRNA cells that shows loss of CFAP57 protein in three of the hTEC cultures. An immunoblot with actin antibody was used as a loading control. (C) Immunofluorescent staining of hTEC with antibodies to acetylated α-tubulin (green) and CFAP57 (magenta) after shRNA-targeted silencing with shRNA2 of *CFAP57*. (D) Ciliary beat frequency of *CFAP57* silenced cells showing altered beat frequency. Each dot represents random area measurement (n = 3 replicates). Scale bar = 10 μm.

To further examine the role of *CFAP57* on ciliary motility, the phenotype of CFAP57 deficient cells was examined by silencing expression using an RNAi approach in hTEC. Primary hTEC were transduced with *a* control plasmid expressing a non-targeted shRNA sequence and a green fluorescent tag [62] or a *CFAP57*-specific shRNA plasmid using a recombinant lentivirus that contains a cassette that confers puromycin resistance [63]. Four different shRNA sequences were screened for CFAP57 knock-down in hTEC. *CFAP57* expression is reduced by three out of the four *CFAP57*-specific shRNA sequences when compared to cells transduced with non-targeted shRNA sequences using qRT-PCR. Two sequences were used for analysis, in three biological replicates using different donor cell lines (Fig 4A and 4A’). Immunoblot analyses confirms the absence of the protein from total cell lysates (Fig 4B), and immunofluorescent staining shows the absence of CFAP57 in ciliated cells (Fig 4C). High-speed video microscopy analysis of ciliary motility [64] of the *CFAP57*-silenced cultures showed significantly reduced CBF when compared to control cells (Fig 4D). Analysis of ciliary beat using high speed video microscopy showed subtle changes in the ciliary waveform, which results in a slightly reduced curvature in *CFAP57*-silenced cells (S3 and S4 Videos) compared to control cells (S5 and S6 Videos).

### CFAP57 is conserved in organisms with motile cilia

To evaluate the conservation of CFAP57 across species, we constructed a phylogenetic tree for CFAP57 (S4 Fig). CFAP57 is found in most organisms with motile cilia, except in the cycads, gingkoes, and the water fern, *Marsilea*. As expected, CFAP57 is missing in organisms that lack motile cilia (flowering plants, nematodes, most fungi) and is missing in *T*. *pseudonana* that lacks inner dynein arms [65]. The N-terminus of the protein is predicted to be composed of WD40 repeats that form beta-sheets and the C-terminus is predicted to form coiled coils and be α-helical [66].

### Effect of *fap57* mutations in *Chlamydomonas*

*Chlamydomonas* is an important model for identifying ciliary genes and probing their functions [67]. To further investigate the function of CFAP57, we carried out additional studies in *Chlamydomonas*. We obtained three insertional mutant strains (LMJ.RY0402.157050, LMJ.RY402.107706 and LMJ.RY0402.211005) from the CLiP collection, which is an indexed library of insertional mutations in *Chlamydomonas* [68, 69]. Insertions in two of the three strains were verified by PCR (S5 Fig). The insertion and drug resistance co-segregate with the swimming phenotype described below in 21 tetrads for LMJ.RY402.107706 and 7 tetrads for LMJ.RY0402.157050. Strain LMJ.RY402.107706 carries an insert in exon 2 and strain LMJ.RY0402.157050 carries an insert in intron 7. We examined the mRNA for the two mutants that had been backcrossed twice and the allele is based on the CLiP name. The transcript in *fap57-706* could not be amplified across exons 2 and 3. In strain *fap57-050*, the transcript for exons 1–7 is present, but we were unable to amplify the remaining exons (S5 Fig). These alleles failed to complement the *bop2-1* allele in diploid strains and are tightly linked to the FAP57 locus (n = 89 tetrads).

### Defective swimming and ciliary waveform in *fap57 Chlamydomonas*

**Fig 5:**
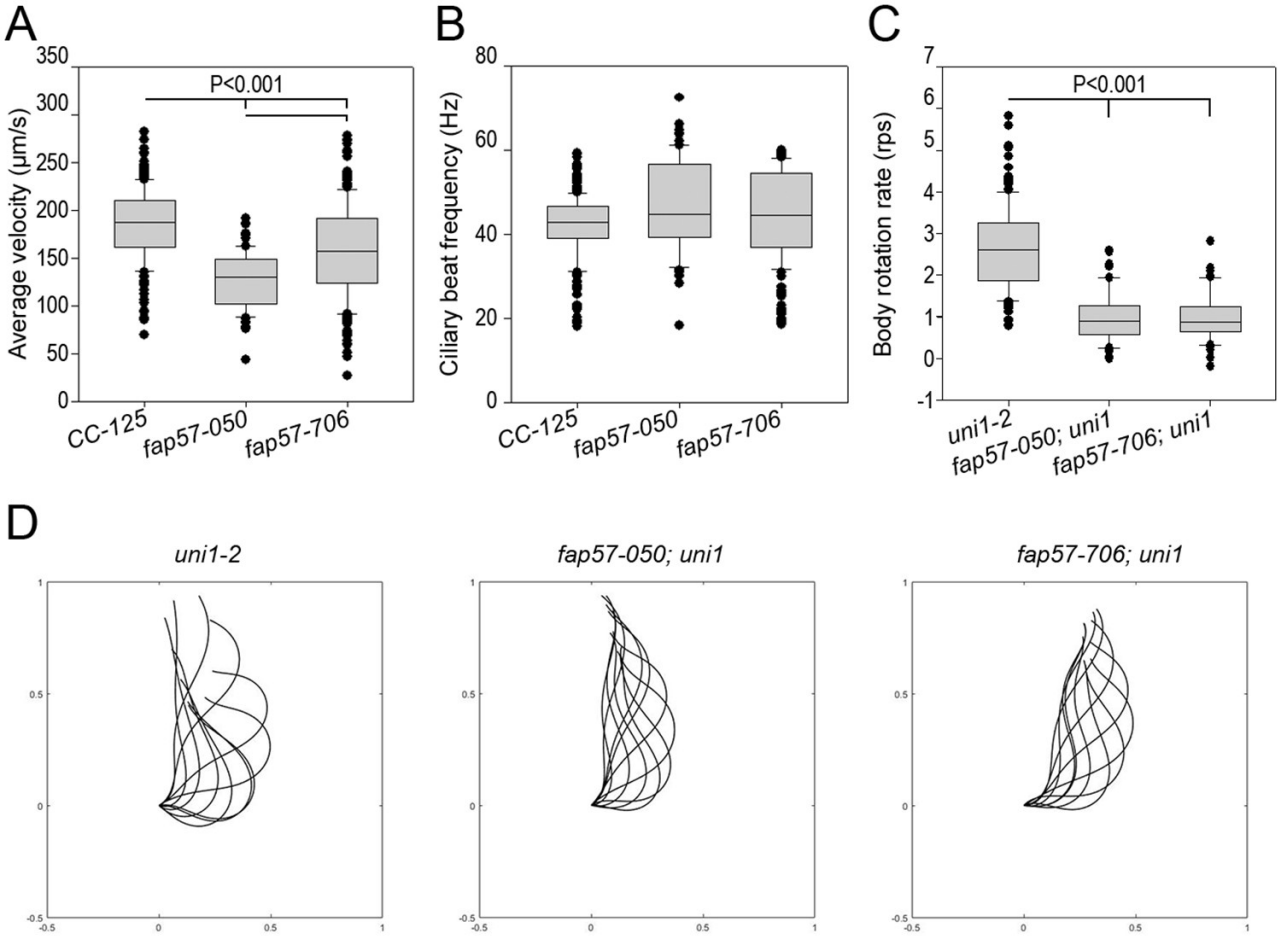
Defective swimming and ciliary waveform in *fap57 Chlamydomonas*. Analysis of swimming behavior of wild-type (CC-125), two *fap57* mutants, and four rescued strains. (A) Average swimming velocity (μm/s). B) Ciliary beat frequency (Hz) extracted from the trajectory. Ciliary waveform analysis of uniciliate wild-type (*uni1-2*) and *fap57* mutants. (C) Cell body average rotation rate (revolutions per second, rps). (D-F) Representative dimensionless (scaled) ciliary waveforms. (D) wild-type (E) *fap57-050* allele (F) *fap57-706* allele.

We analyzed the swimming behavior of these mutants (S2 Table). Swimming velocity analysis shows that *fap57-050* and *fap57-706* swim significantly slower than wild-type cells (CC-125). *fap57-050* was also slower than *fap57-706* (Fig 5A; Table 1). The ciliary beat frequency extracted from the trajectory was unchanged compared to wild-type cells (Fig 5B). Using the *FAP57* gene [52], we transformed *fap57-050*. Two independent transformants were analyzed and both showed wild-type swimming. These transformants were crossed to *fap57-706* and wild-type swimming was also observed for this allele (Table 1).

**Table 1:** Swimming and ciliary beat frequency of *fap57* mutants and rescued strains.

Strain	Average Velocity (μm/s)	Ciliary Beat Frequency (Hz)	Cilia length (μm)	No. of Cells
CC-125 (wild-type)	185.7 ± 37.8	44.3 ± 10.3	11.0 ± 1.9	245
*fap57-050*	127.9 ± 29.4	46.9 ± 10.8	11.1 ± 2.1	88
*fap57-706*	157.8 ± 48.9	44.7 ± 11.0	10.7 ± 2.0	205
*fap57-050; FAP57TG-1*	175.7 ± 27.8	44.3 ± 10.3	11.2 ± 1.9	50
*fap57-050; FAP57TG-2*	182.7 ± 31.8	46.3 ± 9.8	11.0 ± 1.7	50
*fap57-706; FAP57TG-1*	179.7 ± 32.4	45.3 ± 11.7	10.5 ± 1.6	50
*fap57706; FAP57TG-2*	180.7 ± 17.8	42.3 ± 12.1	10.9 ± 1.8	50

To characterize the waveform of mutants, both the *fap57-050* and *fap57-706* mutants were crossed with the uniciliate mutant *uni1-2* [70]. Fifty movies of *fap57-050; uni1* and 51 movies of *fap57-706; uni1* were compared to 153 movies of *uni1-2* (the wild-type group was previously described by Bottier *et al*. [71]) (Fig 5A, S6 Fig). Body motion analysis shows that both mutants have a significantly slower body rotation rate compared to wild-type cells (Fig 5C), which agrees with the reduced velocity. Both *fap57* strains have a significantly reduced bend amplitude (S6B Fig) and an increased average curvature compared to wild-type cells (S6C Fig). The *fap57-050* mutants also have an average curvature significantly smaller than *fap57-706*, which suggests that the *fap57* mutant waveforms are less wavy, and straighter. Both the amplitude of power and torque are significantly reduced for the *fap57* mutants compared to wild-type (S6D Fig). Those results are compatible with a reduced swimming velocity as well as a reduced body net rotation. Both alleles displayed waveforms with a shorter stroke. Differences between the two strains may be explained by the different mRNAs that are made in the two strains.

#### Protein composition of *fap57* mutant cilia show a partial phenotype

Using isobaric tags (TMT) for tandem mass spectroscopy, we analyzed isolated cilia from four wild-type strains and two technical replicates of two different *fap57* meiotic progeny. There was an average of 107 FAP57 peptides in wild-type cilia, but only 6 peptides in mutant cilia, which suggests a strong loss of function of both alleles (Table 2). Several of the inner dynein arm heavy chains (DHC7, 3, 2) are reduced (Table 2) compared to the other dynein heavy chains (S3 Table). The translational elongation factor 1 alpha (EEF1A1) is also reduced in the *fap57* mutant strains to the same extent as the inner dynein arms. This elongation factor is present in proteomic analyses of both *Chlamydomonas* and human axonemes [43, 58, 72–74], which suggests it is not a cytoplasmic contaminant and it is likely to have a ciliary role. It can be extracted from axonemes with KCl and is also found in the membrane matrix fraction [43]. Four additional proteins are significantly reduced but have few spectral counts (Table 2). Cre12.g540050 and Cre10.g438500 have no orthologs outside of the green algae. Cre13.g562800 has a paralog in *Chlamydomonas* (Cre07.g313850) and both are likely orthologs of WDR49. Cre10.g438500/FAP264 is an ortholog of LRRC74. In humans, WDR49 is expressed in the fallopian tubes and lung and FAM74A is highly expressed in the testes based on the GTEx project [75]. FBB7 is increased in our four mutant samples as well as in the dataset of Lin et al. [52].

**Table 2:** TMT proteomics on wild-type and *fap57* mutants.

Protein/Gene	No. Peptides	*Fold change relative to all samples*		
		Average of wild-type^a^	*fap57-1*^b^	*fap57-2*^b^
FAP57 Cre04.g217917	107	1.0	0.1/0.1	0.1/0
DHC7; **g** Cre06.g265950	341	0.98	0.5/0.5	0.6/0.5
DHC3; minor **g** Cre06.g265950	78	0.95	0.5/0.5	0.5/0.6
DHC2; **d** Cre09.g392282	540	1.0	0.8/0.8	0.8/0.8
Translational elongation factor 1α Cre06.g263450	220	0.98	0.3/0.6	0.6/0.6
Cre12.g540050	5	1.05	0.3/0.3	0.3/0.3
Fbox/WD repeat Cre13.g562800	5	1.0	0.3/0.3	0.3/0.3
CCDC89 Cre03.g174800	4	0.92	0.6/0.7	0.5/0.6
FAP264 Cre10.g438500	3	0.9	0.3/0.3	0.3/0.2
FBB7 Cre03.g143827	27	0.95	2.0/2.0	2.0/1.3

^**a**^ the average of two axonemal preparations from CC-125 and two axonemal preparations from CC-4533.

^b^ the ratio for each of mutant strain to the wild-type strains is presented

### *fap57* insertional mutants suppress the *pf10* mutant

The *FAP57* gene maps near the *BOP2* locus and the phenotype of the *fap57* insertional mutants is similar to the *bop2-1* mutant strain [51]. The *bop2-1* mutation results in slow swimming and the loss of inner dynein arms on a subset of the doublets [51, 52]. Since *bop2-1* was isolated as a suppressor of the motility defect of the *pf10* mutant, we tested if the *fap57-706* and *fap57-050* alleles suppress the motility defect of *pf10*. Both alleles acted as suppressors as measured by the number of cells that can oppose gravity in liquid medium (S4 Table).

Taken together, the above data demonstrate that FAP57 plays an important role in the assembly of a specific subset of IDAs, and the proper positioning of these IDAs is important for the generation of normal waveforms. This is consistent with the phenotype observed in the PCD subject, in which absence of CFAP57 results in reduced CBF and altered waveform in the absence of a structural defect detectable by TEM.

## Discussion

Mutations in over 40 genes have been reported to cause PCD [2, 27]. However, many cases of PCD (~30%) remain unsolved with no known genetic cause. The large number of causative genes is a direct reflection of the complexity of ciliary assembly and structure. Many of the known mutations occur in genes that encode structural proteins of the ODAs or preassembly factors for ODAs and IDAs. To date, no mutations have been reported that cause a specific defect in only the IDAs in humans. We performed whole exome capture and sequencing on a patient with a clinical diagnosis of PCD who had no known genetic mutation, and no obvious structural changes visible by TEM. Using PSAP, we identified an apparently homozygous nonsense mutation in *CFAP57*. The PSAP algorithm uses large population sequencing databases and pathogenicity prediction algorithms to calculate the probability of sampling a particular genotype, or set of genotypes, observed in a single n = 1 case. PSAP is most useful in rare disease studies where multiple families are unavailable, the cohort size is modest (dozens to hundreds), and the disease presents with genetic heterogeneity. In the other patients with no TEM phenotype, no additional variants in *CFAP57* were identified. PSAP is being used to examine cases of idiopathic nonobstructive azoospermia to identify new causative genes [76]. It is likely that PSAP will become a useful tool to identify causative genetic variants for rare diseases. These may include diseases similar to azoospermia and PCD that are characterized by large effect mutations and extensive locus heterogeneity. In cultured nasal epithelial cells from the PCD patient, we found that the sequence variant in CFAP57 resulted in the skipping of the exon 11. This suggests that the c.1762C>T change may disrupt an exonic splice enhancer, which results in the production of a mutant protein. The short protein is localized at the basal body region, but fails to assemble onto the ciliary axoneme. Exon 11 encodes the last WD repeat of CFAP57; WD repeats play roles as scaffolds in mediating protein interactions. The intraflagellar transport machinery (IFT) is composed of more than 20 proteins that are assembled into two complexes called A and B. The B complex is thought to carry most of the ciliary cargo via weak interactions [77]. The IFT-B proteins contain both WD repeats and TPR proteins that are thought to be important for interactions with cargo. Cargo loading onto IFT has been studied in *Chlamydomonas*. Tubulin, which is the major IFT cargo, has a dedicated binding site on two of the IFT proteins [78]. ODA transport require ODA16 (NP_849143) as an assembly factor and the N-terminus of IFT46 for transport [79–81]. The two-headed inner dynein arm I1/f has an adaptor (IDA3) that is needed for transport of the I1/f two headed dynein and the associated intermediate and light chains [82]. It is possible that the 58 amino acids missing in the PCD patient may be important for the loading of CFAP57 onto the intraflagellar transport machinery (IFT) for its transport from the cytoplasm to the cilia. Because the immunofluorescence for CFAP57 protein was performed on ciliated cells treated with detergent, which permeabilizes the cell membrane and results in the loss of IFT trains, we do not know if the defect is loading. We cannot rule out the possibility that the short CFAP57 is defective in association with the axoneme rather than in transport. Future studies can distinguish between these possibilities.

Cilia lacking CFAP57 exhibit a significantly reduced CBF and an altered waveform. Knockdown of *CFAP57* in hTEC cells using shRNA results in a reduction of mRNA and protein that was confirmed by immunofluorescence and immunoblotting. These cells show a reduced CBF. In contrast, TEM analysis of nasal epithelial cells from the subject appear normal and immunostaining for the ODA protein DNAH5 was unchanged. Immunostaining for DNALI1 also did not show significant differences between control and PCD-II samples. DNALI1 (p28 in *Chlamydomonas*) is not reduced in the proteomic analysis of the *Chlamydomonas* mutants. This is not surprising as it is associated with inner dynein arm a and c as well as d in *Chlamydomonas* [83]. The d dynein in *Chlamydomonas* associates uniquely with two proteins. They are p38 and p44 and their human orthologs are ZMYND12 and TTC29, respectively. Both are present in the proteome of human motile cilia. Analysis of these two proteins may be a useful way to screen for dynein d mutants when antibodies become available.

Although the mechanisms that regulate basal CBF have been studied extensively, they are still unclear. These results suggest that a proper balance between ODAs and IDAs is also required to maintain normal CBF in human airway cells. Waveform analysis revealed subtle differences between the PCD cells and controls that varied among the cells. The heterogeneous waveform observed between individual cilia in the PCD samples could be related to the maturity of individual cilium. It has been reported that variations in ciliary waveform are associated with the progression of ciliary length and the differentiation of ciliated cells [84].

Although the ciliary axoneme is described as showing nine-fold symmetry, there are many structural asymmetries in the cilium [85]. The generation of waveforms requires the spatial and temporal regulation of dyneins, and this is likely to require structural asymmetries. Multiple approaches have helped to catalog the asymmetric structures and proteins in *Chlamydomonas* cilia. These asymmetries have been identified through analysis of mutant *Chlamydomonas* flagella by electron microscopy and proteomics and are beginning to provide a wealth of information to use for understanding how asymmetric and symmetric waveforms are generated and propagated [47, 48, 86]. If all of the ciliary dyneins were active at one time, the cilia would be in a rigor state, resulting in no net movement or bending. In order to generate an effective bend, dynein motor function must be tightly controlled both along the length of the cilium and around the circumference of the axoneme across a defined axis; dynein motors on one side of the axis are active during the effective stroke, while the dyneins on the opposite side of the axis become active during the recovery stroke [87]. The asymmetrical features are likely to be key to generate the waveforms.

The EM analysis of the *Chlamydomonas bop2-1* mutant showed that FAP57 is required for the assembly of dynein arms on only a subset of the nine doublet microtubules [51]. In recent work using cryo-EM tomography, there is a loss of a subset of inner dynein arms on doublet microtubules 5–8 with a partial loss on doublets 1 and 9 [52]. Lin et al. provides data that FAP57 forms an extended filament that connects to several structures. The proteomic analysis of *fap57* mutants suggests that dynein d (DHC7) and g (DHC2) are reduced in the mutant along with the minor dynein, DHC3, which assembles at the far end of the 96 nm repeat in the proximal region where dynein g is found distally and could be considered the proximal g inner dynein (Table 2, [52]). The loss of these dyneins on only a subset of doublet microtubules is sufficient to affect the swimming velocity and the waveform in *Chlamydomonas*. The CFAP57 filament may act like the CCDC39/CCDC40 ruler that specifies the addresses of the N-DRC and radial spokes [88, 89]. Finding the protein(s) that is needed on the other doublets will be an important next step.

Examination of the *Chlamydomonas* genome identified a paralog of FAP57, which is FBB7. The FBB7 protein (Cre03.g143827/PNW84378.1) like FAP57 has multiple WD40 repeats in the N-terminus although it is predicted to be about 400 amino acids longer. It is present in the published *Chlamydomonas* ciliary proteome [43] as well as in our sample. The message is upregulated by deciliation [40]. FBB7 is ~ 24% identical and 41% similar to both *Chlamydomonas* FAP57 and human CFAP57, but FAP57 is 41% identical and 60% similar to human CFAP57. There is an average of a 1.8-fold increase in the number of FBB7 peptides in the *fap57* mutants (Table 2; S7 Fig), which agrees with the results of Lin et al [52]. This is similar to the findings for the tether/tether head paralogs, FAP43 and FAP244 in *Chlamydomonas* [90, 91]. FAP43 is missing from the distal one-fifth of the axoneme in wild-type samples. In the *fap244* mutant, FAP43 localizes along the entire length of the axoneme [91]. The FAP43 protein appears to be able to dock at the “address” for FAP244 in the *fap244* mutant. It will be interesting to test if FBB7 is found on doublets 1–4 in wild-type and the other doublets in the *fap57* mutant. Since humans have no paralog of CFAP57, we speculate that some inner dynein arms will be lost from all of the doublets and not just a subset as in *Chlamydomonas*.

FAP43 and FAP44 are required for the formation of the tether and tether head (T/TH) complex, which is required for the positional stability of the I1/f dynein motor domains, as well as the stable anchoring of CK1 kinase, and proper phosphorylation of the regulatory IC138 subunit in *Chlamydomonas*. Interestingly, T/TH also interacts with the inner dynein arm d and radial spoke 3 [90]. In *Tetrahymena*, CFAP57 is placed adjacent to the FAP43/44 complex based on proximity mapping [92]. Comparative proteomics analysis of I1/f and FAP43/44 mutants showed that the I1 dynein and the T/TH complex assemble independently of each other [90]. The *fap57* mutants assemble the Il/f two-headed dynein complex properly as well (S3 Table) based on our proteomic data.

In comparison to *Chlamydomonas*, less is known about the detailed structure of the IDA in human cilia. Based on the high level of conservation between species, it is likely that CFAP57 plays a similar role in human cilia as it does in *Chlamydomonas*. However, because the planar waveform of human cilia is inherently different from the waveforms of *Chlamydomonas*, the exact positioning and regulation of IDA activity is also likely different. Additional studies using advanced techniques to culture and study human respiratory cells are needed to address these questions.

In summary, our results show that a genetic variant in *CFAP57* creates an in-frame deletion that disrupts the localization of the protein to the axoneme. The absence of CFAP57 reduces CBF and alters waveform, which results in PCD. Based on the analysis of mutants in *Chlamydomonas*, it is likely due to defective assembly of a subset of IDA. This report is the first example of PCD caused by mutation of a protein that appears to affect only a subset of the IDAs. The short mutant CFAP57 protein suggests that we have identified an important domain that can be studied to understand the assembly of IDAs. These findings also demonstrate the usefulness of the PSAP algorithm and set a precedent to consider it in the evaluation of other cases of PCD with no obvious structural defects. Identifying the genetic basis of PCD and the functional defects is an important step toward developing personalized treatments for this rare disease.

## Methods

### Ethics statement

The individuals included in this research study provided informed consents and all protocols involving human studies were approved by the University of North Carolina Medical School Institutional Review Board.

### Human genetic analysis

A cohort of 99 PCD patients with no obvious EM phenotype was assembled for research exome sequencing. Exome libraries were prepared using an IDT capture reagent. Genetic variants were discovered and genotyped using a validated analysis pipeline at the Washington University McDonnell Genome Institute as previously described [56]. Each case was analyzed using the population sampling probability (PSAP) framework, a published statistical method for identifying pathogenic mutations from n = 1 cases of rare disease [54]. Segregation analysis was performed on the available DNA from family members (family UNC-1095). The primers used are listed in S5 Table.

### Airway epithelial cell cultures

Human nasal epithelial (HNE) cells from the PCD subject (proband 2-II) and unrelated healthy controls were obtained as described [93]. The nasal cells were expanded as conditionally reprogrammed cells (CRC) by co-culturing them with irradiated 3T3 fibroblasts plus the RhoA kinase inhibitor Y-27632 [59] and differentiated as previously described [33].

Human tracheobronchial epithelial cells (hTEC) were obtained from non-smoking donors lacking respiratory pathologies provided by the Cystic Fibrosis Center Tissue Procurement and Cell Culture Core [57], or were isolated from surgical excess of tracheobronchial segments of lungs donated for transplantation as previously described [18]. These unidentified cells are exempt from regulation by HHS regulation 45 CFR Part 46. hTEC cells were expanded *in-vitro* and allowed to differentiate using air-liquid interface (ALI) conditions on supported membranes (Transwell, Corning Inc., Corning, NY), as previously described [18, 57]. These protocols have been approved by the University of North Carolina (UNC) Medical School Institutional Review Board.

### shRNA for silencing of *CFAP57*

shRNA sequences were obtained from Sigma-Aldrich in pLK0.1 lentivirus vector, and were chosen from the Broad institute RNAi library. Target sequences evaluated included (Sequence 1: CCTGGAACTATTTAAGGAATA, Sequence 2: CAGAAAGTAATGGCCATTGTT, sequence 3: CCCTGGAACTATTTAAGGAAT, sequence 4: CCTTCCATTCACCCTTCTCAT, sequence 5: GCCTAGGAAATCATCAGAGA).

### Analysis of *CFAP57* expression

RNA was isolated from cells using an RNeasy Mini Kit (Qiagen) and RT-PCR was performed using specific primers (S5 Table) as previously described [33]. For qRT-PCR, RNA was reverse-transcribed using an Applied Bioscience High-Capacity Reverse Transcription Kit (Thermo Fisher Scientific). Gene expression was detected by RT-PCR using gene specific primers, (FW:AAAGCAGAACTGTTTGGCGG, RV:TTGGGACTGATGGACAAGGC) and a SYBR green nucleic acid-labeling SYBR FAST kit (Kapa Biosystems) in a Lightcycler 480 (Roche). Fold change was calculated using the delta–delta cycle threshold [ΔΔC(t)] analysis method and OAZ1 as an internal control. For RNAseq analysis, hTEC cells were cultured using ALI conditions, and sampled at ALI day 7, 14, and 21. Three samples from each time point were used for analysis. RNA was extracted using a Qiagen RNeasy kit. RNA library preparation and analysis were performed by the Washington University Genome Center (GTAC), using Clontech-SMARTer RNAseq kit, and sequenced using an Illumina Hiseq3000 sequencer, for a total depth reads of at least 40 million reads per sample.

For *Chlamydomonas* RNA isolation, cells from two R medium agar plates grown for 5 days were resuspended in 40 ml nitrogen-free medium (M-N/5) for 2 h at room temperature to allow flagellar assembly. The cells were then collected, and RNA extraction was performed with the RNeasy Mini Kit (Qiagen) according to the manufacturer’s recommendation. Two micrograms of RNA was used in a reverse transcription reaction with SuperScript III (Invitrogen) with random primers as previously described [94].

### Epithelial cell immunofluorescence

Airway cells were fixed and immunostained as previously described [58, 95, 96]. The binding of primary antibodies was detected using Alexa Fluor-488, Alexa flour-555, Alexa Fluor-647 (Life Technologies), indocarbocyanine (CY3) or Rhodamine Red-X (RRX) conjugated secondary antibody (Jackson ImmunoResearch Laboratories, West Grove, PA). The DNA was stained using 4’, 6-diamidino-2-phenylindole (DAPI, Vector Laboratories, Burlingame, CA, USA) or with Hoechst 33342 FluoroPure (Life Technologies). Slides were mounted using ProLong Diamond antifade mountant (Thermo Fisher). Images were acquired using a Zeiss -710 microscopy system or a Leica epifluorescent microscope (LAS X, Leica, Buffalo Grove, IL). Images were processed and the fluorescence intensity analyzed using FIJI [97] as previously described [58]. Brightness and contrast were adjusted globally using Photoshop (Adobe Systems, San Jose, CA). Isotype matched control antibodies had no detectable staining under the conditions used. The antibodies used are listed in S6 Table.

### Ciliary isolation, protein extraction and immunoblot

Ciliary isolation and protein extraction was performed as previously described [58]. Protein extraction from tissue or cells and immunoblot analysis was performed as previously described [58, 95].

### Transformation of *Chlamydomonas*

To generate rescued strains, *Chlamydomonas* cells were transformed as previously described with a NEPA21 square-pulse electroporator [98, 99] in two independent experiments with 250 ng of *FAP57* plasmid DNA and 250 ng of *APHVIII* plasmid DNA. 500 colonies were picked and scored for the presence of the *FAP57* plasmid by PCR. 78 colonies contained both ends of the gene and 54 of these showed rescue of the swimming phenotype by microscopic examination.

### *Chlamydomonas* axoneme isolation

*Chlamydomonas* cilia were isolated using the dibucaine method [100]. Cilia were demembranated with the addition of 1% Nonidet P-40 in HMDS-EGTA (10 mM HEPES, 5 mM MgSO_4_, 1 mM DTT, 4% Sucrose, 0.5 mM EGTA, pH7.4) buffer and separated from the membrane and matrix fractions by centrifugation. Isolated cilia were resuspended in HMDEK (30 mM HEPES, 5 mM MgSO_4_ 1 mM DTT, 0.5 mM EGTA, 25 mM KCl, pH 7.45) buffer. 100 micrograms of proteins from each sample were digested by trypsin and given isobaric tags before undergoing mass spectrometry (Donald Danforth Plant Science Center, St. Louis, MO).

### Tandem mass spectroscopy

The samples were lysed in lysis buffer (8M Urea in 50mM HEPES pH 8.0) and sonicated briefly. Samples were reduced with 10 mM TCEP and alkylated with 25 mM iodoacetamide. Protein concentrations were determined by BCA protein assay (Thermo Scientific). Fifty μg of protein was taken from each sample for digestion. Sequencing grade protease Lys-C was added with 1:250 ratio and sample was digested overnight at room temperature with mixing. A second digestion was performed by diluting the sample with 50 mM HEPES to lower the urea concentration to 1M and trypsin was added with 1:100 ratio for a further 12 hour digestion at room temperature with mixing. Digests were acidified with formic acid and subjected to Oasis HLB solid phase extraction column (Waters).

### Tandem Mass Tag (TMT) Labeling

Digested peptides were labeled according to the TMT 10plex reagent kit instructions. Briefly, TMT regents were brought to room temperature and dissolved in anhydrous acetonitrile. Peptides were labeled by the addition of each label to its respective digested sample. Labeling reactions were incubated without shaking for 1 h at room temperature. Reactions were terminated with the addition of hydroxylamine. Subsequent labeled digests were combined into a new 2 mL microfuge tube, acidified with formic acid, subjected to Sep-Pak C18 solid phase extraction and dried down.

### High pH reverse phase fractionation

The dried peptide mixture was dissolved in 110 μL of mobile phase A (10 mM ammonium formate, pH 9.0). 100 μL of the sample was injected onto a 2.1 x 150 mm XSelect CSH C18 column (Waters) equilibrated with 3% mobile phase B (10 mM ammonium formate, 90% ACN). Peptides were separated using a gradient [101] to at a flow rate of 0.2 mL/min. 60 peptide fractions were collected corresponding to 1 min each. 10 pooled samples were generated by concatenation in which every 10th fraction (1, 11, 21, 31, 41, 51; six fractions total) was combined. The 10 pooled samples were acidified and dried down prior to LC-MS analysis.

#### LC-MS analysis

Each fraction was resuspended in 50 μL 1% acetonitrile/1% formic acid. 5 μL was analyzed by LC-MS (HCD for MS/MS) with a Dionex RSLCnano HPLC coupled to a Velos Pro OrbiTrap mass spectrometer (Thermo Scientific) using a 2h gradient. Peptides were resolved using 75 μm x 25 cm PepMap C18 column (Thermo Scientific).

#### Data analysis

All MS/MS samples were analyzed using Proteome Discoverer 2.1 (Thermo Scientific). The Sequest HT search engine in the Proteome Discover was set to search *Chlamydomonas* database (Uniprot.org). The digestion enzyme was set as trypsin. The HCD MS/MS spectra were searched with a fragment ion mass tolerance of 0.02 Da and a parent ion tolerance of 20 ppm. Oxidation of methionine was specified as a variable modification, while carbamidomethyl of cysteine and TMT labeling was designated at lysine residues or peptide N-termini were specified in Proteome Discoverer as static modifications.

MS/MS based peptide and protein identifications and quantification results was initially generated in Proteome Discover 2.1 and later uploaded to Scaffold (version Scaffold_4.8.2 Proteome Software Inc., Portland, OR) for final TMT quantification and data visualization. Normalized and scaled protein/peptide abundance ratios were calculated against the abundance value of the four wild-type controls. Peptide identifications were accepted if they could be established at greater than 80.0% probability by the Peptide Prophet algorithm [102] with Scaffold delta-mass correction. Protein identifications were accepted if they could be established at greater than 99.0% probability and contained at least 2 identified peptides. Protein probabilities were assigned by the Protein Prophet algorithm [103]. Proteins that contained similar peptides and could not be differentiated based on MS/MS analysis alone were grouped to satisfy the principles of parsimony. Proteins sharing significant peptide evidence were grouped into clusters.

#### Preparation of *Chlamydomonas* for video microscopy

*Chlamydomonas* cells were grown as previously described [104]. Cells were grown on agar plate for 48 hours in Sager and Granick rich liquid medium [105] at 25°C in constant light. Prior to recording, cells were suspended for 3 hours in a medium lacking nitrogen adapted from Medium I of Sager and Granick [105] to promote gametogenesis. Cells were directly recorded under the microscope.

All bright field microscopy was carried in a climate-control room maintained at 21°C. For each recording, 10 μL from liquid cells culture (after gametogenesis) were pipetted onto a slide and a cover slip (18 x 18 mm) was placed for recording under a Zeiss Axiophot (Carl Zeiss AG, Oberkochen, Germany) with a 40x Plan-Neofluar objective lens for swimming analysis and with 100x Neofluar oil-immersion objective lens for waveform analysis. Videos were recording using a Phantom Miro eX2 camera and Phantom Camera Control Application 2.6 (Vision Research, Inc, Wayne, NJ, USA). Videos were captured at 2000 frames per second with 320 x 240 resolution and an exposure time of 200 μs. Around 7000 frames with 3500 frames before the trigger and 3500 frames after the trigger were captured. Frames displaying a characteristic beating/swimming were extracted and saved under uncompressed AVI format at 15 frames per second.

#### Ciliary beat frequency and waveform analysis

The ciliary beat frequency (CBF) in HNE cultures (n = 6) was measured as previously described [33, 106]. In hTEC cultures, CBF was measured in at least 5 fields obtained from each preparation. The cultures were maintained at 37°C using a temperature controller and a stage heater block. High-speed videos (120 frames/s) were recorded and processed with the Sisson-Ammons Video Analysis system (SAVA, Amons Engineering, Mt Morris, MI) as described [18, 107]. To analyze the ciliary waveform, cells were lifted off the supportive membranes and imaged directly on slides. To evaluate the waveform and beat direction, high resolution videos of ciliated cells were recorded at 200 fps using a 60X Plan-Apo oil objective (NA = 1.4), DIC optics, and ambient temperature. High resolution videos of 6 cells in 3 different cultures were recorded as previously described [33]. The videos were analyzed by an experienced scientist blinded to the genotype of the cells. Videos were replayed in slow motion. The ciliary length was measured and the waveform of the front and back cilia in 4 ciliated cells was traced manually. Deviation from linearity was determined by measuring the furthest ciliary displacement from the linear axis as defined by the end-effective and end-recovery position, as previously described [61].

#### *Chlamydomonas* swimming analysis

Each video were analyzed using ImageJ [108] to create a binary file only displaying the cells. Each pixel had a spatial resolution of 310 x 310 nm and the temporal resolution between 2 consecutive time points was 1 ms. Cells were tracked using the 2D/3D single-particle tracking tool of the MosaicSuite for ImageJ and Fiji (MOSAIC Group, MPI-CBG, Dresden). Custom-made program written in Matlab R2016a (The Mathworks, Natick, MA, USA) was then used to compute the cells velocity as well as the ciliary beat frequency extracted by Fast-Fourier-Transform of the trajectory.

#### *Chlamydomonas* kinematic analysis of cilium

The uniciliate mutant strain *uni1-2* and the double mutants *fap57-050; uni1* and *fap57-706; uni1* were generated from meiotic crosses, as described by Dutcher [109]. The single mutant *uni1* is considered the wild-type reference as previously published [70]. Videos were analyzed using a custom-made program written in Matlab R2016a (The Mathworks, Natick, MA, USA) previously published [71]. From each video, a sequence of 200 consecutive frames was stored in a 3D matrix of pixel intensity values. Each pixel had a spatial resolution of 169 x 169 nm and the temporal resolution between 2 consecutive time points was 0.5 ms. Components of the forces exerted by cilia on the fluid are directly calculated from the Cartesian coordinates using parameters previously reported [71].

#### Statistical analyses

Group variation is described as mean ± standard error (SEM). Statistical comparisons between groups were made using 1-way analysis of variance (ANOVA) with Tukey post-hoc analysis. Individual group differences were determined using a 2-tailed Student’s t-test. A p value of 0.05 was considered to represent a significant difference. Non-parametric data are shown as the median and the 25^th^ and 75^th^ intraquartile ranges. Data were analyzed using Prism (GraphPad, LaJolla, CA).

## Supporting information

S1 Fig CFAP57 in non-cultured cells. Images showing examples of CFAP57 immunostaining in non-cultured normal tracheal ciliated cells. Cells were scraped off fresh human trachea, immunostained for detection of acetylated alpha-tubulin and CFAP57 as detailed in the Materials and Methods. Scale bar 10 μm.
(DOCX)

S2 Fig RT-PCR analysis of subject mRNA. Example image of the entire gel evaluating the presence and size of the transcript of *CFAP57* in PCD proband 2-II and unrelated control cells. DNAI1, a ciliary specific gene was used as a control of normal ciliogenesis and PPIA as a housekeeping gene control.
(DOCX)

S3 Fig Immunofluorescence analysis of axonemal components in PCD 2-II cells. The staining of isolated ciliated cells from control and PCD 2-II cultures showed normal distribution of (A) DNAH5 (n = 3 cells control and PCD) and (B) RSPH1 (n = 3 PCD cells; n = 4 control) in different regions of interest (ROI: proximal, middle, and tip) of the ciliary axoneme. The corresponding graph shows the corrected total cilia fluorescence (CTCF) in each ROI. C) Analysis of the fluorescence intensity for DNALI1 in isolated human nasal ciliated cells obtained from a control (n = 5 cells) and the PCD 2-II subject (n = 13 cells). These data suggest a reduction of the localization of DNALI1 at the tip of the cilia (p = 0.03).
(DOCX)

S4 Fig Bootstrap analysis of CFAP57. (DOCX)

S5 Fig Molecular analysis of insertional mutants in *Chlamydomonas*. (A) Diagram of *FAP57* genomic DNA with arrows showing the location of the *fap57-050* and *fap57-706* insertions. (B) List of primers used to amplify *CFAP5*7 cDNA and genomic DNA.(C) Locations verified for the LMJ.RY0402.157050 and LMJ.RY0402.107706 insertions by PCR. (D) PCR amplification of regions of *fap57* cDNA. No template indicates that no DNA was added into the reaction.
(DOCX)

S6 Fig Ciliary waveform analysis in *fap57* mutant *Chlamydomonas*. Ciliary beat frequency (Hz) estimated from tangent angle. (B) Bend amplitude (rad). (C) Average curvature (rad/μm). (D) Average torque applied by the cilium about the center of the cell body (pN-μm).
(DOCX)

S7 Fig Density plots of five proteins from TMT mass spectroscopy of wild-type and mutant *Chlamydomonas*. (A). Comparison of peptides from wild-type and mutant peptides for FAP57 (Cre04.g217917). (B). Comparison of peptides from wild-type and mutant peptides for DHC7/dynein g (Cre06.g265950). (C). Comparison of peptides from wild-type and mutant peptides for WDR49 paralog (Cre13.g562800). (D). Comparison of peptides from wild-type and mutant peptides for DHC1; I1/f dynein (Cre12.g48425). (E). Comparisons of peptides from wild-type and *fap57* mutants. One of the *fap57* mutants showed a different level of enrichment, which is responsible for the increased breadth of the curves in this panel. Wild-type strains: Orange: CC-125; Pink CC-4533 (parent of the insertional mutants). Mutant strains: Yellow: *fap57-050*; Blue: *fap57-705*.
(DOCX)

S1 Table List of the top 10 genes identified in the proband using PSAP analysis. (DOCX)

S2 Table Measurements of wild-type and mutant *Chlamydomonas*. (DOCX)

S3 Table TMT values from mass spectroscopy of isolated axonemes. (DOCX)

S4 Table Suppression of the motility defect of *pf10*. (DOCX)

S5 Table Primers used in this study. (DOCX)

S6 Table Antibodies used in this study. (DOCX)

S1 Video S1 Video of control cells. Video recording of control human nasal cells in profile demonstrating normal waveform. Video was recorded at 200 fps using a 60x objective with DIC optics. Playback speed is 15% of normal speed. Scale bar, 4 μm.
(MP4)

S2 Video S2 Video of *cfap57* PCD cells. Video recording of PCD 2-II human nasal cells in profile demonstrating heterogeneous waveform. Video was recorded at 200 fps using a 60x objective with DIC optics. Playback speed is 25% of normal speed. Scale bar, 5 μm.
(MP4)

S3 Video S3 Video of control cells. Control hTEC transduced with a non-targeted shRNA sequence. The movie shows the normal ciliary waveform, at normal speed (S1).
(MOV)

S4 Video S4 Video of control cells. Control hTEC transduced with a non-targeted shRNA sequence. The movie shows the normal ciliary waveform slowed to 1/10x (S2).
(MP4)

S5 Video S5 Video of *CFAP57* silenced cells. Representative images of hTEC transduced with a shRNA to a CFAP57 sequence. The movie shows a reduced curvature at normal speed.
(MOV)

S6 Video S6 Video of CFAP57 silenced cells. Representative images of hTEC transduced with a shRNA to a CFAP57 sequence. The movie shows a reduced curvature slowed to1/10x.
(MP4)

S1 Data Summary of the ciliary beat frequency (CBF) measurements. Measurements from high-resolution videos of control and PCD 2-II ciliated cells to generate the CBF.
(XLSX)

S2 Data Data to determine swimming behavior of wild-type and mutant strains. Measurements used in the analysis of swimming behavior of wild-type (CC-125), and two *fap57* mutants in Fig 5.
(XLSX)

S3 Data Data for proteomics of wild-type and fap57 strains. Peptide data for Table 2, S3 Table and S7 Fig.
(XLSM)
