# Supplementary material for: Mutation of CFAP57, a protein required for the asymmetric targeting of a subset of inner dynein arms in Chlamydomonas, causes primary ciliary dyskinesia
Source: PLoS Genet. 2020 Aug 7;16(8):e1008691. doi: 10.1371/journal.pgen.1008691 (PMC7444499; doi:10.1371/journal.pgen.1008691)
Supplement: S1 Fig — Images showing examples of CFAP57 immunostaining in non-cultured normal tracheal ciliated cells. Cells were scraped off fresh human trachea, immunostained for detection of acetylated alpha-tubulin and CFAP57 as detailed in the Materials and Methods. Scale bar 10 μm. (DOCX) [file pgen.1008691.s001.docx]

**S1 Fig. CFAP57 in non-cultured cells**


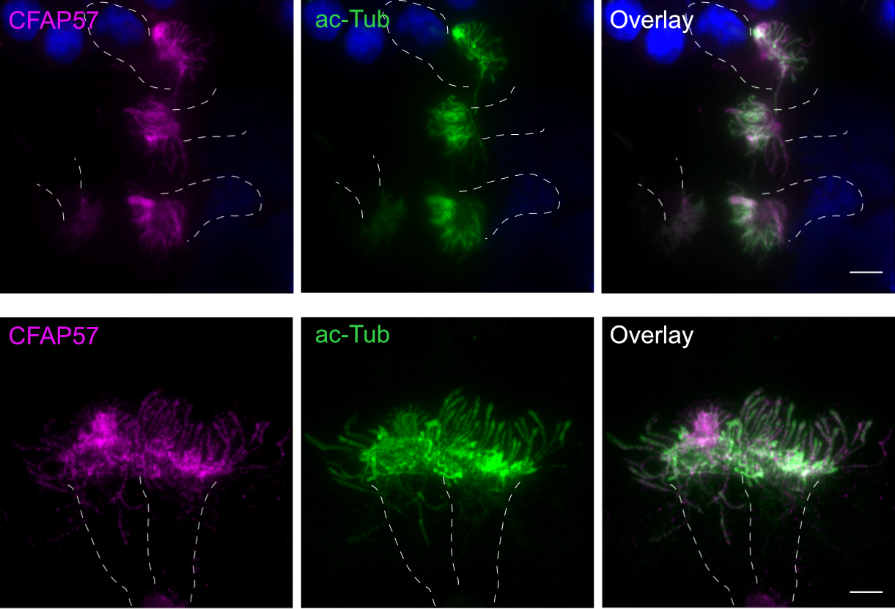


Images showing examples of CFAP57 immunostaining in non-cultured normal tracheal ciliated cells. Cells were scraped off fresh human trachea, immunostained for detection of acetylated alpha-tubulin and CFAP57 as detailed in the Materials and Methods. Scale bar 10 µm.
