# Supplementary material for: Mutation of CFAP57, a protein required for the asymmetric targeting of a subset of inner dynein arms in Chlamydomonas, causes primary ciliary dyskinesia
Source: PLoS Genet. 2020 Aug 7;16(8):e1008691. doi: 10.1371/journal.pgen.1008691 (PMC7444499; doi:10.1371/journal.pgen.1008691)
Supplement: S2 Fig — Example image of the entire gel evaluating the presence and size of the transcript of CFAP57 in PCD proband 2-II and unrelated control cells. DNAI1, a ciliary specific gene was used as a control of normal ciliogenesis and PPIA as a housekeeping gene control. (DOCX) [file pgen.1008691.s002.docx]

**S2 Fig. RT-PCR analysis of subject mRNA**

**
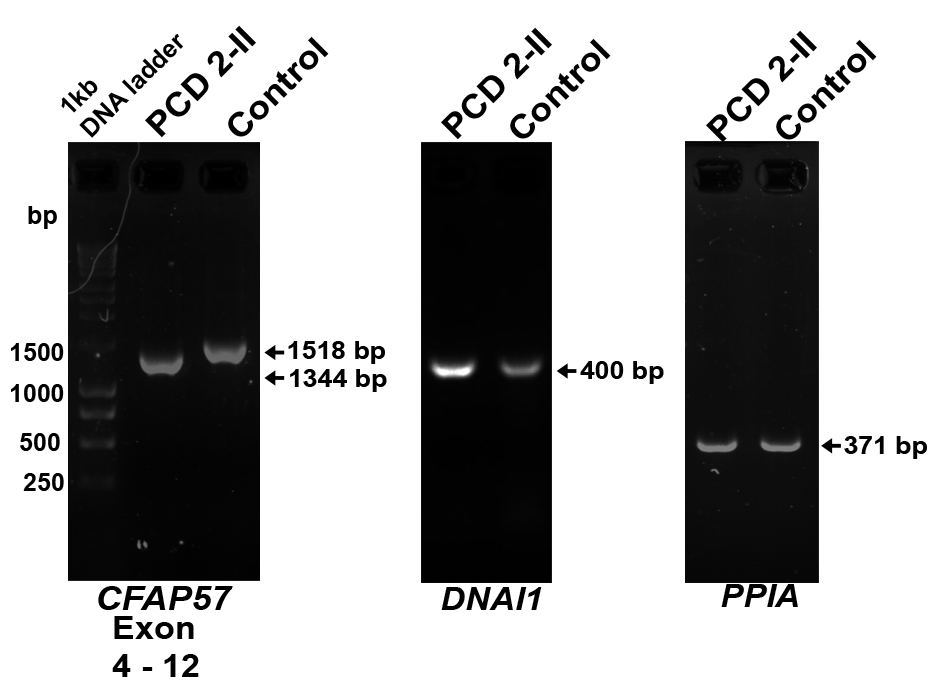
**

Example image of the entire gel evaluating the presence and size of the transcript of *CFAP57* in PCD proband 2-II and unrelated control cells. DNAI1, a ciliary specific gene was used as a control of normal ciliogenesis and PPIA as a ­housekeeping gene control.
