# Supplementary material for: Mutation of CFAP57, a protein required for the asymmetric targeting of a subset of inner dynein arms in Chlamydomonas, causes primary ciliary dyskinesia
Source: PLoS Genet. 2020 Aug 7;16(8):e1008691. doi: 10.1371/journal.pgen.1008691 (PMC7444499; doi:10.1371/journal.pgen.1008691)
Supplement: S3 Fig — The staining of isolated ciliated cells from control and PCD 2-II cultures showed normal distribution of (A) DNAH5 (n = 3 cells control and PCD) and (B) RSPH1 (n = 3 PCD cells; n = 4 control) in different regions of interest (ROI: proximal, middle, and tip) of the ciliary axoneme. The corresponding graph shows the corrected total cilia fluorescence (CTCF) in each ROI. C) Analysis of the fluorescence intensity for DNALI1 in isolated human nasal ciliated cells obtained from a control (n = 5 cells) and the PCD 2-II subject (n = 13 cells). These data suggest a reduction of the localization of DNALI1 at the tip of the cilia (p = 0.03). (DOCX) [file pgen.1008691.s003.docx]

**S3 Fig. Immunofluorescence analysis of axonemal components in PCD 2-II cells**

**
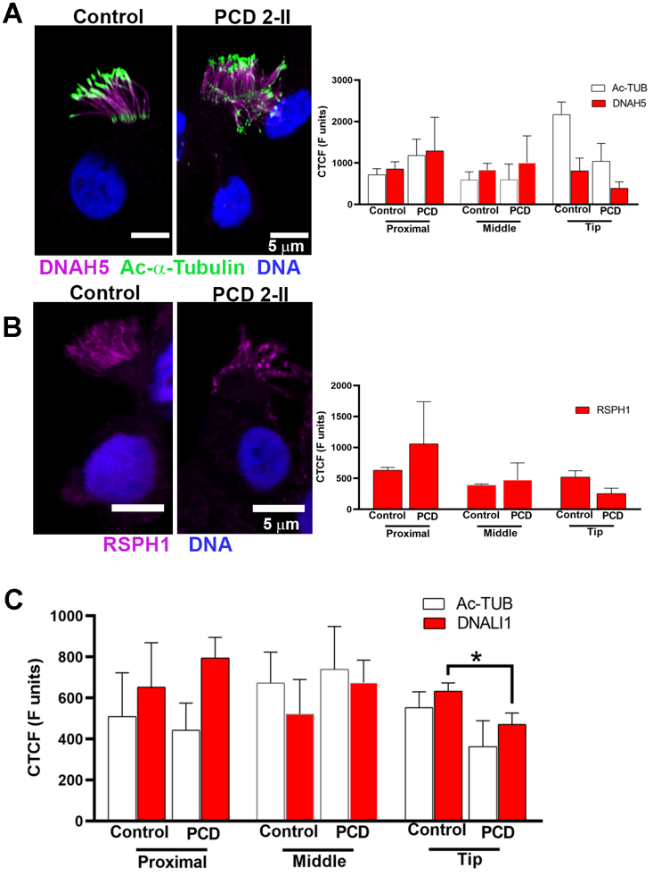
**

The staining of isolated ciliated cells from control and PCD 2-II cultures showed normal distribution of (A) DNAH5 (n=3 cells control and PCD) and (B) RSPH1 (n=3 PCD cells; n=4 control) in different regions of interest (ROI: proximal, middle, and tip) of the ciliary axoneme. The corresponding graph shows the corrected total cilia fluorescence (CTCF) in each ROI. C) Analysis of the fluorescence intensity for DNALI1 in isolated human nasal ciliated cells obtained from a control (n=5 cells) and the PCD 2-II subject (n=13 cells). These data suggest a reduction of the localization of DNALI1 at the tip of the cilia (p = 0.03).
