# Supplementary material for: Mutation of CFAP57, a protein required for the asymmetric targeting of a subset of inner dynein arms in Chlamydomonas, causes primary ciliary dyskinesia
Source: PLoS Genet. 2020 Aug 7;16(8):e1008691. doi: 10.1371/journal.pgen.1008691 (PMC7444499; doi:10.1371/journal.pgen.1008691)
Supplement: S4 Fig — (DOCX) [file pgen.1008691.s004.docx]

**S4 Fig. Bootstrap analysis of CFAP57**


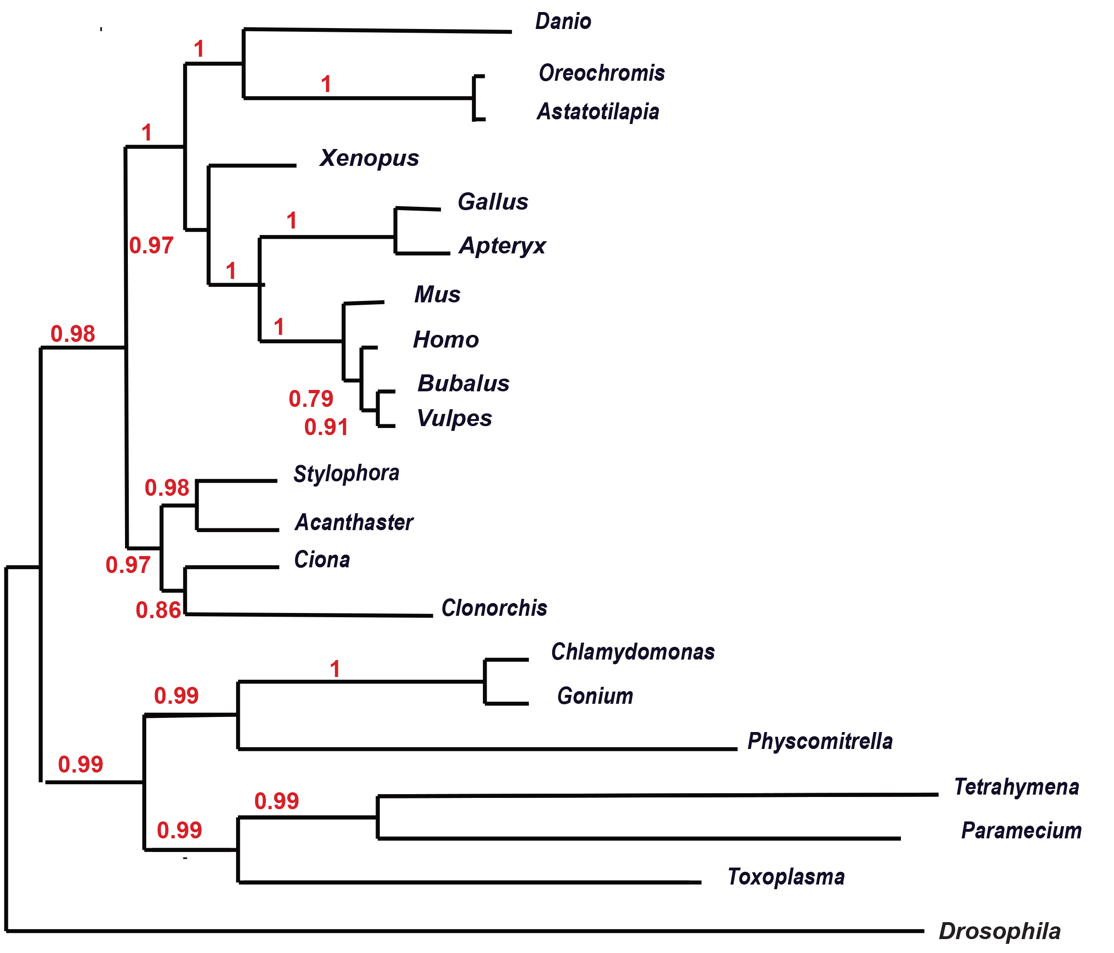


**Reference**

1. Dereeper A*, et al.* (2008) Phylogeny.fr: robust phylogenetic analysis for the non-specialist. *Nucleic acids research* 36(Web Server issue):W465-469.
