# Supplementary figures and images for: Mutation of CFAP57, a protein required for the asymmetric targeting of a subset of inner dynein arms in Chlamydomonas, causes primary ciliary dyskinesia
Source: PLoS Genet. 2020 Aug 7;16(8):e1008691. doi: 10.1371/journal.pgen.1008691 (PMC7444499; doi:10.1371/journal.pgen.1008691)

**S5 Fig. Molecular analysis of insertional mutants in *Chlamydomonas***


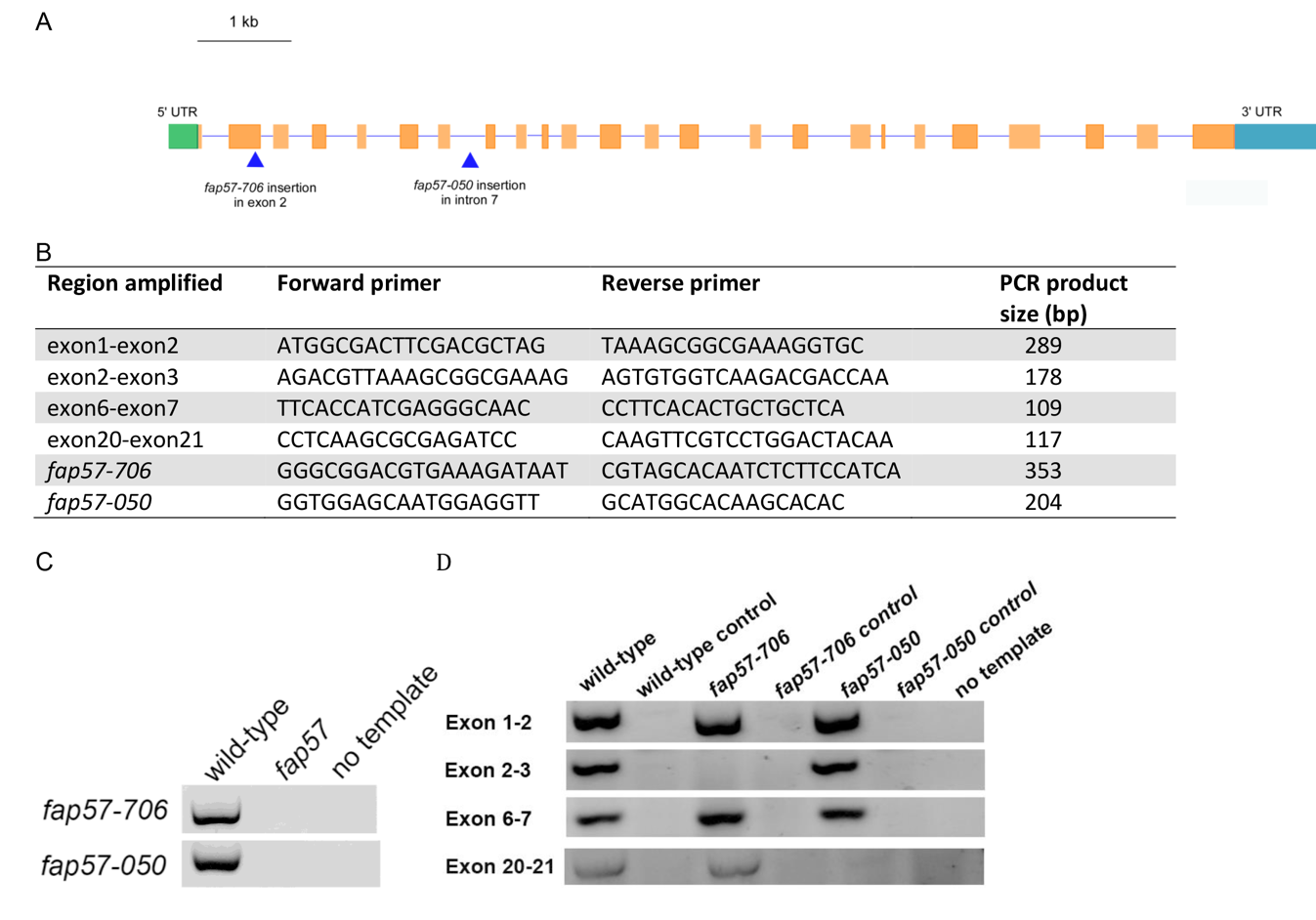

Supplement: S5 Fig — (A) Diagram of FAP57 genomic DNA with arrows showing the location of the fap57-050 and fap57-706 insertions. (B) List of primers used to amplify CFAP57 cDNA and genomic DNA.(C) Locations verified for the LMJ.RY0402.157050 and LMJ.RY0402.107706 insertions by PCR. (D) PCR amplification of regions of fap57 cDNA. No template indicates that no DNA was added into the reaction. (DOCX) [file pgen.1008691.s005.docx]
