# Supplementary material for: Mutation of CFAP57, a protein required for the asymmetric targeting of a subset of inner dynein arms in Chlamydomonas, causes primary ciliary dyskinesia
Source: PLoS Genet. 2020 Aug 7;16(8):e1008691. doi: 10.1371/journal.pgen.1008691 (PMC7444499; doi:10.1371/journal.pgen.1008691)
Supplement: S6 Fig — Ciliary beat frequency (Hz) estimated from tangent angle. (B) Bend amplitude (rad). (C) Average curvature (rad/μm). (D) Average torque applied by the cilium about the center of the cell body (pN-μm). (DOCX) [file pgen.1008691.s006.docx]

**S6 Fig. Ciliary waveform analysis in *fap57* *Chlamydomonas***

**
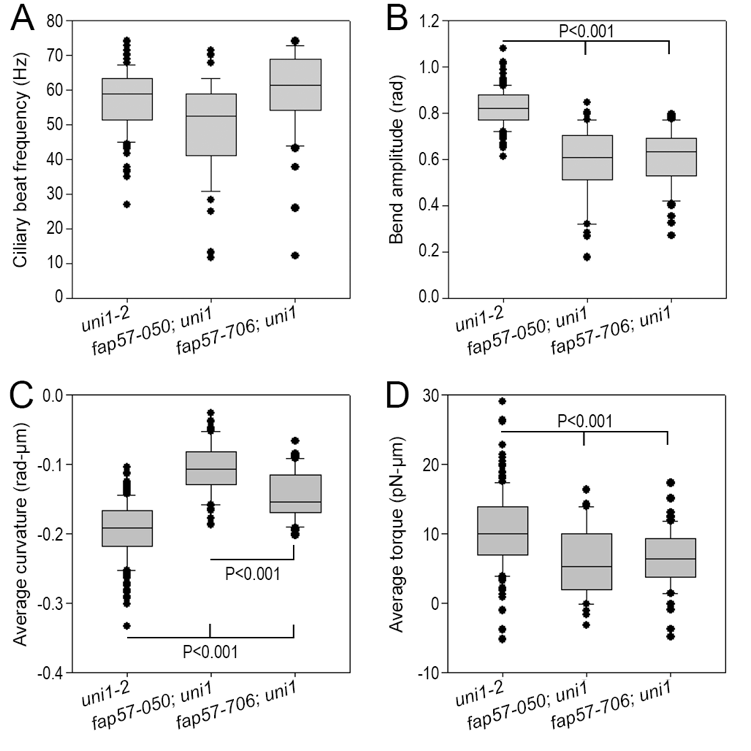
**

(A) Ciliary beat frequency (Hz) estimated from tangent angle. (B) Bend amplitude (rad). (C) Average curvature (rad/µm). (D) Average torque applied by the cilium about the center of the cell body (pN-µm).
