# Supplementary material for: Mutation of CFAP57, a protein required for the asymmetric targeting of a subset of inner dynein arms in Chlamydomonas, causes primary ciliary dyskinesia
Source: PLoS Genet. 2020 Aug 7;16(8):e1008691. doi: 10.1371/journal.pgen.1008691 (PMC7444499; doi:10.1371/journal.pgen.1008691)
Supplement: S7 Fig — (A). Comparison of peptides from wild-type and mutant peptides for FAP57 (Cre04.g217917). (B). Comparison of peptides from wild-type and mutant peptides for DHC7/dynein g (Cre06.g265950). (C). Comparison of peptides from wild-type and mutant peptides for WDR49 paralog (Cre13.g562800). (D). Comparison of peptides from wild-type and mutant peptides for DHC1; I1/f dynein (Cre12.g48425). (E). Comparisons of peptides from wild-type and fap57 mutants. One of the fap57 mutants showed a different level of enrichment, which is responsible for the increased breadth of the curves in this panel. Wild-type strains: Orange: CC-125; Pink CC-4533 (parent of the insertional mutants). Mutant strains: Yellow: fap57-050; Blue: fap57-705. (DOCX) [file pgen.1008691.s007.docx]

**S7 Fig. Density plots of four proteins from TMT mass spectroscopy of wild-type and mutant *Chlamydomonas*
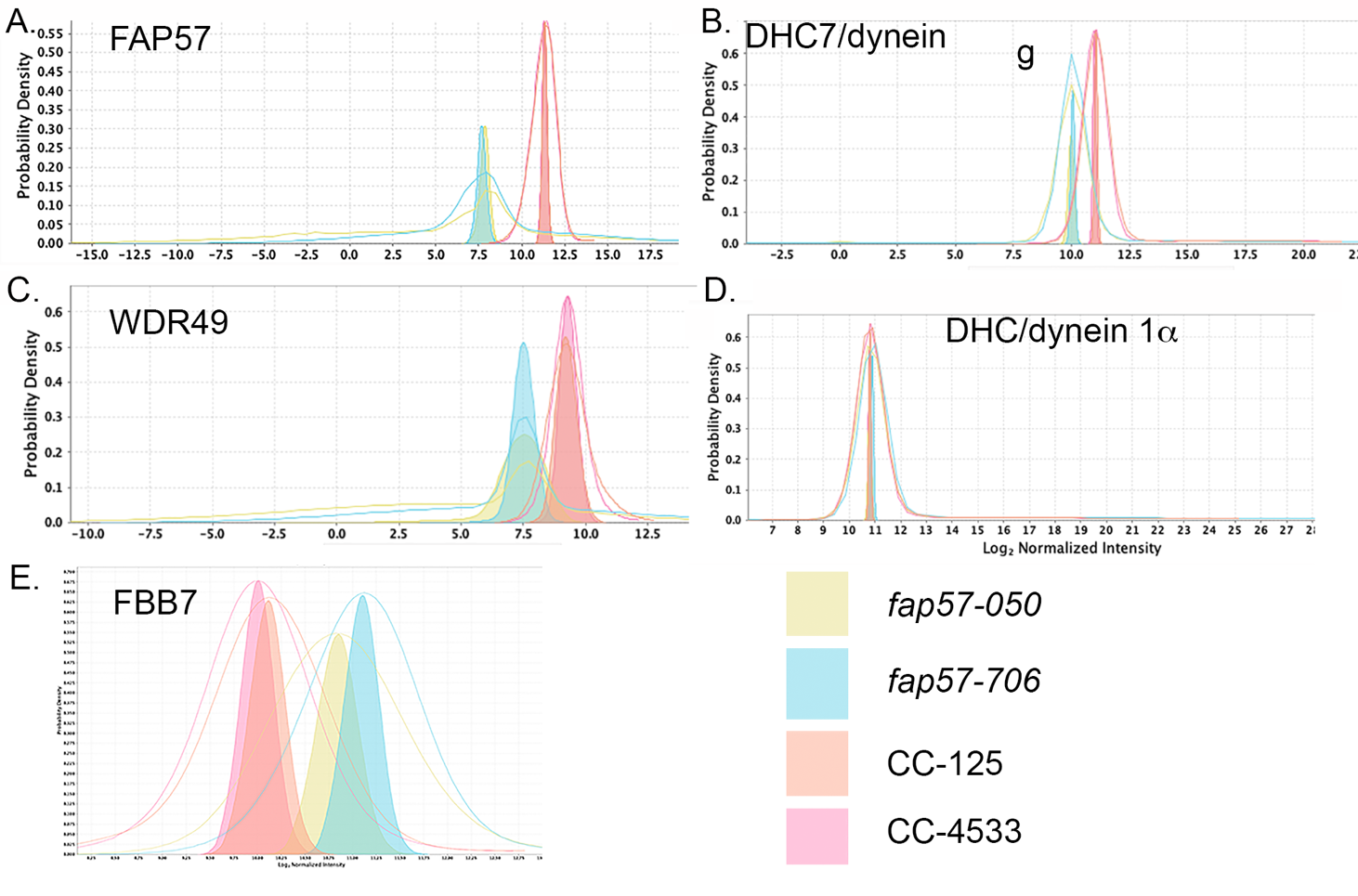
**

**(**A). Comparison of peptides from wild-type and mutant peptides for FAP57 (Cre04.g217917) . (B). Comparison of peptides from wild-type and mutant peptides for DHC7/dynein g (Cre06.g265950). (C). Comparison of peptides from wild-type and mutant peptides for WDR49 paralog (Cre13.g562800). (D). Comparison of peptides from wild-type and mutant peptides for DHC1; I1/f dynein (Cre12.g48425). (E). Comparisons of peptides from wild-type and *fap57* mutants. One of the *fap57* mutants showed a different level of enrichment, which is responsible for the increased breadth of the curves in this panel. Wild-type strains: Orange: CC-125; Pink CC-4533 (parent of the insertional mutants). Mutant strains: Yellow: *fap57-050*; Blue: *fap57-705.*
