# Supplementary material for: Mutation of CFAP57, a protein required for the asymmetric targeting of a subset of inner dynein arms in Chlamydomonas, causes primary ciliary dyskinesia
Source: PLoS Genet. 2020 Aug 7;16(8):e1008691. doi: 10.1371/journal.pgen.1008691 (PMC7444499; doi:10.1371/journal.pgen.1008691)
Supplement: S1 Table — (DOCX) [file pgen.1008691.s008.docx]

S1 Table. List of the top 10 genes identified in the proband using PSAP analysis.

| **Gene** | **Transcript** | **Position (hg19)** | | **Type** | **AA Change** | **exAC Freq** | **CADD** | **PSAP pvalue** |
| --- | --- | --- | --- | --- | --- | --- | --- | --- |
| *CD2* | ENST00000369478.3 | 1:117311391_C>T | nonsynonymous SNV | | P348S | 0.0015 | 17.22 | 2.00E-06 |
| *WDR65/CFAP57* | ENST00000372492.4 | 1:43675420_C>T | stopgain | | R588X | 1.72E-05 | 37 | 3.00E-06 |
| *ETS1* | ENST00000392668.4 | 11:128350323_A>T | nonsynonymous SNV | | F296I | 3.00E-04 | 25.7 | 5.00E-06 |
| *TMOD4* | ENST00000416280.2 | 1:151143016_T>C | nonsynonymous SNV | | M263V | 0.0046 | 13.71 | 1.20E-05 |
| *TEX15* | ENST00000256246.2 | 8:30695552_C>T | nonsynonymous SNV | | E2367K | 9.19E-05 | 16.87 | 1.30E-05 |
| *CEND1* | ENST00000330106.4 | 11:788386_G>C | nonsynonymous SNV | | S64W | 2.60E-05 | 10.56 | 1.80E-05 |
| *CTU2* | ENST00000453996.2 | 16:88780568_C>T | nonsynonymous SNV | | R344W | 1.67E-05 | 18.85 | 2.00E-05 |
| *HSD3B1* | ENST00000528909.1 | 1:120054192_G>T | nonsynonymous SNV | | R71I | 0.0039 | 11.87 | 2.50E-05 |
| *CCDC74B* | ENST00000310463.6 | 2:130902418_A>G | nonsynonymous SNV | | L51P | 1.00E-04 | 13.63 | 2.70E-05 |
| *GUCY2F* | ENST00000218006.2 | X:108638614_C>T | nonsynonymous SNV | | E794K | 0.0042 | 27.4 | 5.70E-05 |
|  |  |  |  | |  |  |  |  |

**Supplemental Table 1**. List of the top 10 genes identified in the proband using PSAP analysis. The “PSAP” p-value is a measure of significance or “surprise” of sampling a particular genotype. It represents the probability of sampling a genotype given a population genetic model trained on a large database of “normal” control individuals. Smaller p-values are more surprising or unlikely (2, 3)
