# Supplementary material for: Mutation of CFAP57, a protein required for the asymmetric targeting of a subset of inner dynein arms in Chlamydomonas, causes primary ciliary dyskinesia
Source: PLoS Genet. 2020 Aug 7;16(8):e1008691. doi: 10.1371/journal.pgen.1008691 (PMC7444499; doi:10.1371/journal.pgen.1008691)
Supplement: S2 Table — (DOCX) [file pgen.1008691.s009.docx]

**S2 Table.** **Measurements of wild-type and mutant *Chlamydomonas***

|  | ***uni1-2*** | ***fap57-050; uni1-2*** | ***fap57-706; uni1-2*** |
| --- | --- | --- | --- |
| **Body rotation**  **Rotations/sec** | 2.6 ± 1.0 | 1.0 ± 0.6 | 1.0 ± 0.6 |
| **Beat frequency (Hz)** | 57.4 ± 8.6 | 49.6 ± 13.5 | 59.1 ± 12.5 |
| **Bend amplitude**  **(rad/µm)** | 0.82 ± 0.08 | 0.59 ± 0.16 | 0.61 ± 0.12 |
| **Average curvature**  **rad/µm** | -0.20 ± 0.04 | -0.11 ± 0.04 | -0.14 ± 0.04 |
| **Force**  **(pN/µm)** | 10.5 ± 5.8 | 5.8 ± 5.6 | 6.3 ± 4.3 |
| **Internal forces**  **(pN/µm)** | 193 ± 42 | 188 ± 56 | 187 ± 53 |
