# Supplementary material for: Mutation of CFAP57, a protein required for the asymmetric targeting of a subset of inner dynein arms in Chlamydomonas, causes primary ciliary dyskinesia
Source: PLoS Genet. 2020 Aug 7;16(8):e1008691. doi: 10.1371/journal.pgen.1008691 (PMC7444499; doi:10.1371/journal.pgen.1008691)
Supplement: S3 Table — (DOCX) [file pgen.1008691.s010.docx]

**S3 Table. TMT values from mass spectroscopy of isolated axonemes**

|  |  | ***Log_2_ Fold Change*** | | |  |
| --- | --- | --- | --- | --- | --- |
| **Protein/Gene** | **No.**  **Peptides** | **Wild-type**  **Average of wild-type**  **(n=4)** | ***fap57-1***  **replicates** | ***fap57-2***  **replicates** | ***Quantitized***  ***Spectral Count*** |
| **Dynein arms that are reduced in mutants** | | | | | |
| DHC7; **g**  Cre06.g265950 | 341 | -0.025 | -0.9/-1.1 | -0.8/-1.3 | 222 |
| DHC2; **d**  Cre09.g392282 | 540 | 0 | -0.3/-0.4 | -0.4/-0.4 | 358 |
| DHC3 (minor IDA)  Cre06.g265950 | 78 | -0.075 | -1.0/-1.0 | -0.9/-1.1 | 43 |
| **Dynein arms that are not reduced in *fap57* mutants** | | | | | |
| ODA11; DHC13; α  Cre11.g476050 | 1662 | 0.025 | 0.1/0.2 | 0.1/0 | 1124 |
| ODA4; DHC14; β  Cre09.g403800 | 1471 | 0 | 0.2/0.2 | 0.1/0.1 | 490 |
| ODA2; DHC15; γ  Cre01.g058400 | 1443 | 0.025 | 0.1/0.2 | 0.1/0 | 345 |
| DHC6; **a**  Cre05.g244250 | 444 | 0.05 | 0.2/0.2 | 0.2/-0.1 | 285 |
| DHC5; **b**  Cre02.g107050 | 342 | 0 | 0.2/0.2 | 0.1/0 | 202 |
| DHC9; **c**  Cre02.g141606 | 609 | -0.05 | 0.1/0.1 | 0/0 | 199 |
| DHC8; **e**  Cre16.g685450 | 346 | -0.025 | 0.2/0.2 | 0.1/0 | 211 |
