# Supplementary material for: Mutation of CFAP57, a protein required for the asymmetric targeting of a subset of inner dynein arms in Chlamydomonas, causes primary ciliary dyskinesia
Source: PLoS Genet. 2020 Aug 7;16(8):e1008691. doi: 10.1371/journal.pgen.1008691 (PMC7444499; doi:10.1371/journal.pgen.1008691)
Supplement: S4 Table — (DOCX) [file pgen.1008691.s011.docx]

**S4 Table. Suppression of the motility defect of *pf10***

| **Genotype** | **Ratio of cells in supernatant to total cell number**  **(n= 300)** |
| --- | --- |
| *FAP57; pf10* | 0.1 |
| *FAP57; PF10* | 0.97 |
| *fap57-050; pf10* | 0.94 |
| *fap57-706; pf10* | 0.95 |
