# Supplementary material for: Mutation of CFAP57, a protein required for the asymmetric targeting of a subset of inner dynein arms in Chlamydomonas, causes primary ciliary dyskinesia
Source: PLoS Genet. 2020 Aug 7;16(8):e1008691. doi: 10.1371/journal.pgen.1008691 (PMC7444499; doi:10.1371/journal.pgen.1008691)
Supplement: S5 Table — (DOCX) [file pgen.1008691.s012.docx]

**S5 Table. Antibodies used in this study**

| **Antibody** | **Antigen** | **Source** | **Dilution**  **Immunoblot** | **Dilution**  **IF** |
| --- | --- | --- | --- | --- |
| HPA028623 rabbit polyclonal | CFAP57 | Sigma-Aldrich,  St. Louis, MO | 1:1000 | 1:500 |
| HPA036225 rabbit polyclonal | WDR49 | Sigma-Aldrich,  St. Louis, MO | 1:500 | 1:200 |
| 6-11B-1, monoclonal  T7451 | Acetylated α−tubulin | Sigma-Aldrich,  St. Louis, MO | NT | 1:1000 |
| HPA053129  rabbit polyclonal | DNALI1 | Sigma-Aldrich,  St. Louis, MO | NT | 1:500 |
| 5600 | RSPH1 | Abmart, Berkeley Heights, NJ | NT | 1:250 |
| Mouse monoclonal | DNAI1 | University of North Carolina | NT |  |
| Alexa Fluor-488 Secondary antibody |  | Life Technologies, Carlsbad, CA |  |  |
| Alexa Fluor-647  Secondary antibody |  | Life Technologies,  Carlsbad, CA |  |  |
| indocarbocyanine (CY3 conjugated secondary antibody |  | Jackson ImmunoResearch Laboratories, West Grove, PA |  |  |
| Rhodamine Red-X (RRX) conjugated secondary antibody |  | Jackson ImmunoResearch Laboratories, West Grove, PA |  |  |

**Antibodies that failed to produce a ciliary signal in human airway cells**.

| **Antibody** | **Antigen** | **Source** | **Dilution**  **Immunoblot** | **Dilution**  **IF** |
| --- | --- | --- | --- | --- |
| HPA051759  Lot R67114 | EEF1A1 | Sigma-Aldrich,  St. Louis, MO | NT | 1:100 |
| HPA035094 | LRRC74A | Sigma-Aldrich,  St. Louis, MO | NT | 1:100 |
| HPA036225 | WDR49 | Sigma-Aldrich,  St. Louis, MO | 1:500 | 1:200 |
| HPA037006  Lot R33948 | TTC29 | Sigma-Aldrich,  St. Louis, MO | NT | 1:100 |
| ZMYND1212 | ZMYND12 | Biorbyt, Cambridge England | NT | 1:100 |
