# Supplementary material for: Mutation of CFAP57, a protein required for the asymmetric targeting of a subset of inner dynein arms in Chlamydomonas, causes primary ciliary dyskinesia
Source: PLoS Genet. 2020 Aug 7;16(8):e1008691. doi: 10.1371/journal.pgen.1008691 (PMC7444499; doi:10.1371/journal.pgen.1008691)
Supplement: S6 Table — (DOCX) [file pgen.1008691.s013.docx]

**S6 Table. Primer sequences for analysis of *CFAP57***

| **Primer used for amplification of**  **genomic DNA** | | |
| --- | --- | --- |
| **Position** | **Primer Sequence (5’ to 3’)** | **Name** |
| Exon 11 | GAT GCC AAA AGG GGA TAG A | M13 tag sense primer |
|  | CAG GGC TAT GGC TCC TTT C | M13 tag antisense primer |
| **Primers used for amplification of cDNA** | | |
| **Gene** | **Primer Sequences (5’ to 3’)** | **PCR product size (bp)** |
| *CFAP57* | GGG GAG AAC CCC CAA CCA TAT CT  TGA TGG GTG CTG AGT GCA AT | 597 |
| *DNAI1* | AGA GAA GGA GAA GGC AAA GAC CCC  TGT ACT CAG GGA AGC TGG GGT TCT | 400 |
| *PPIA* | CCG TGT TCT TCGACA TTG CC  ACA CCACAT GCT TGC CAT CC | 371 |
